# An urgent need for HIV testing among men who have sex with men and transgender women in Bamako, Mali: Low awareness of HIV infection and viral suppression among those living with HIV

**DOI:** 10.1371/journal.pone.0207363

**Published:** 2018-11-12

**Authors:** Avi J. Hakim, Kelsey Coy, Padmaja Patnaik, Nouhoum Telly, Tako Ballo, Bouyagui Traore, Seydou Doumbia, Maria Lahuerta

**Affiliations:** 1 Division of Global HIV and Tuberculosis, Centers for Disease Control and Prevention (CDC), Atlanta, Georgia, United States of America; 2 Rollins School of Public Health, Emory University, Atlanta, Georgia, United States of America; 3 ICAP at Columbia University, Mailman School of Public Health, New York, New York, United States of America; 4 International Center of Excellence in Research (ICER-Mali), Faculty of Medicine and Odontostomatology, University of Sciences, Techniques and Technology of Bamako (USTTB), Bamako, Mali; 5 Cellule Sectorielle de Lutte contre le Sida, Ministère de la Santé, Bamako, Mali; 6 Department of Epidemiology, Mailman School of Public Health, Columbia University, New York, New York, United States of America; International AIDS Vaccine Initiative, UNITED STATES

## Abstract

Despite the high HIV prevalence among men who have sex with men (MSM) and transgender women (TGW), there are limited data on progress on their respective HIV antiretroviral treatment (ART) cascades to identify progress and gaps in meeting UNAIDS 90-90-90 targets. We conducted a respondent-driven sampling survey of MSM and TGW in Bamako, Mali from October 2014 to February 2015. We describe the HIV treatment cascade for MSM and TGW, identify correlates of being unaware of HIV-infected status and having unsuppressed viral load levels, and estimate proportion of recent infections. We enrolled 387 MSM and 165 TGW. HIV prevalence was 13.7%. Of those living with HIV, 10.4% were aware of their serostatus, 61.2% of them self-reported being on treatment, and of them, 100% were virally suppressed. In multivariate analysis, factors associated with being unaware of HIV infection included not using free condoms in the last six months (aOR: 5.7, 95% CI: 1.1–29.5) and not having comprehensive knowledge of HIV (aOR: 6.5, 95% CI: 1.4–29.9). Having unsuppressed viral load was associated with identifying as a transgender woman (aOR: 4.8, 95% CI: 1.1–20.7) and not having comprehensive knowledge of HIV (aOR: 6.5, 95% CI: 1.0–40.9). Of the 79 HIV-positive participants, 5.1% had recent infections. While the proportion aware of their HIV status was low despite adjusting for viral load biomarkers, all MSM and TGW on treatment were virally suppressed. Improved testing strategies are urgently needed to achieve the first 90 of the HIV cascade among MSM and TGW in Bamako.

## Introduction

The disproportionately high HIV prevalence among men who have sex with men (MSM) and transgender women (TGW) compared to the general population has been well documented.[1–3] Low HIV testing coverage among MSM and TGW suggest that similar disparities may exist throughout the entire HIV cascade.[4–6] Recent years have seen an increase in surveys among MSM in sub-Saharan Africa though few studies include transgender women. These surveys report primarily on HIV prevalence and risk behaviors.[7] Present knowledge of HIV and ways to combat the epidemic necessitate that surveys go beyond measuring these standard variables and expand to include awareness of HIV status, antiretroviral therapy (ART) status, and viral load suppression as articulated by the UNAIDS 90-90-90 targets.[8, 9]

HIV cascade and incidence data are becoming increasingly available for the general population, but remain virtually non-existent for key populations in the published literature.[10–14] There are limited cascade data on all populations but particularly key populations. In addition, estimates of incidence or the proportion of HIV infections in key populations that are recent are virtually nonexistent. Where data on ART coverage and viral suppression do exist, they usually come from cohorts of people already diagnosed with HIV or in care rather than the population at large.[15, 16] Such data do not provide information about HIV incidence, the proportion of people living with HIV who have been diagnosed, or the proportion of diagnosed people not in care or on ART. They may also be prone to the Hawthorne effect (observation bias), which presents a partial and biased view of the problem.[17] Given the challenges and limitations of collecting data on key populations in clinic settings and in general population surveys, biobehavioral surveys (BBS) are an effective tool for illuminating the HIV cascade for key populations, including MSM and TGW.[18] Where sample sizes or data measures allow, they may also provide important information about HIV incidence or the proportion of infections that are recent, both of which play a critical role in characterizing the epidemic.

We conducted a respondent-driven sampling survey of MSM and TGW in Bamako, Mali to facilitate an evidence-based response to Mali’s HIV epidemic. HIV prevalence is estimated at 1.2% in Mali and 130,000 people are believed to be living with HIV.[19] Since 2010 new infections have increased by 11%.[19] HIV prevalence among MSM in Bamako is estimated at 13.7%, eight times the prevalence among general population men in the same city.[20] Homosexuality is not criminalized in Mali; however, stigma and shame may drive many MSM and transgender women to have female sex partners or wives.[21] Elsewhere, these same forces result in poor health outcomes for MSM and TGW compared to the general population.[22] We describe the HIV cascade for MSM and TGW in Mali, identify correlates of being unaware of HIV-infected status and not having suppressed viral load, and estimate proportion of infections that are recent among these populations.

## Methods

### Recruitment

We used respondent-driven sampling to recruit people who were biologically male and have sex with men in Bamako, Mali between October 2014 and February 2015. To participate in the survey, individuals needed to be born biologically male, engaged in anal or oral sex with a man in the past six months, aged ≥ 18 years, been a resident of Bamako or its suburbs in the past six months, speak French or Bambara, be able to provide written informed consent, and be in possession of a valid recruitment coupon. As described elsewhere, recruitment began with six purposively selected seeds of diverse characteristics.[20] One seed was added part way through implementation.

### Survey procedures

Participants underwent a face-to-face interview covering demographics, sexual history, condom and lubricant use, stigma and harassment, violence, and uptake of HIV services. The questionnaire can be found in supporting information (S1 and S2 Appendices). They then received HIV counseling prior to rapid testing according to Mali’s national HIV testing algorithm. Participants with a reactive Determine rapid test (Alere, MA, USA) result received confirmatory testing with the Clearview rapid test (Alere, MA, USA). OraQuick ADVANCE Rapid HIV-1/2 Antibody Test (OraSure Technologies, Inc, PA, USA) was used as a tie breaker in the case of discordant results. Dried blood spots (DBS) were collected for quality control. All participants received post-test counseling, condoms, lubricants, and information about HIV services. Those testing HIV-positive were referred to treatment facilities that had been identified as providing non-stigmatizing services to MSM. Staff at these facilities were trained to receive survey participants and received additional sensitization about the needs of MSM.

HIV-positive specimens were tested for HIV viral load at CDC Atlanta with the Roche Cobas AmpliPrep/Cobas TaqMan (Noblesville, Indiana). Specimens with viral load>1,000 were tested for recency using the LAg-Avidity EIA (Sedia, Sedia Biosciences, Portland, Oregon).

Participants were compensated 4,000 CFA (about US$8) for transportation and time during the first visit. At the second visit they received an additional 1,000 CFA (about US$2) for each successful recruit (up to three) and 2,000 CFA (about US$4) for transport.

### Data analysis

The outcomes of interest were i) being infected with HIV but unaware and ii) not having suppressed viral load. Data were analyzed with RDS-Analyst (RDS-A) (version 0.52, Los Angeles, California, USA) using Gile’s Successive Sampling Estimator (SSE). As RDS-A is unable to estimate confidence intervals for values of 100.0%, Wilson score bounds based on the raw counts were used to estimate uncertainty around viral suppression.

All data presented are adjusted population estimates unless otherwise indicated. Using weights imported from (RDS-A), survey logistic procedures were used in SAS 9.4 (Cary, NC). Variables were considered for inclusion in the model based on the published literature, and those significant at the 0.1 level in the bivariate analysis were included in the multivariate analysis. Using a manual backward stepwise procedure, variables were retained if significant at the 0.05 level based on the Type 3 Analysis of Effect. Variables included in the selection procedure for being HIV-positive and unaware of HIV status were gender identity, problematic consumption of alcohol (AUDIT-C score of at least 4), use of free condoms in the last 6 months, and comprehensive HIV knowledge. Transgender woman was defined as self-identification of being born male and living as a woman at the time of the survey. Variables included in the selection procedure for not being virally suppressed were income, gender identity, social cohesion, problematic consumption of alcohol, having a condom break during anal sex with a man in the last 6 months, ability to get condoms when needed, and comprehensive HIV knowledge. Adjusted odds ratios (aOR) and their 95% confidence intervals (CI) are presented.

A sensitivity analysis was conducted to explore the variation in the cascade between self-reported results for awareness of HIV status and treatment status, and results adjusted whereby anyone who was virally suppressed was also considered to be aware they are living with HIV and on ART. Data were analyzed assuming that anyone with suppressed viral load (<1,000 copies/mL) was also aware of their HIV infection and on treatment. Chi-squared tests were performed to detect differences in awareness of HIV status and viral load suppression. Viremia was defined as viral load>1,500 copies/mL.

Recent infections were defined as testing positive with the limiting-antigen (LAg) avidity assay and not being virally suppressed.

### Ethics approvals

This survey was approved by the Malian Ethical Committee of the Facility of Medicine, Pharmacy and Dentistry, the CDC Associate Director of Science in the Center for Global Health, the Columbia University Medical Center Institutional Review Board, and as a research activity involving human subjects. No personal identifying information were collected as part of this survey.

## Results

We enrolled 387 MSM and 165 TGW, for a total of 552 people. Nearly 40% of coupons (608 of 1,551) were returned to the survey site and 90.8% of people with coupons were eligible to participate in the survey. All but two participants consented to HIV testing.

**Fig 1:**
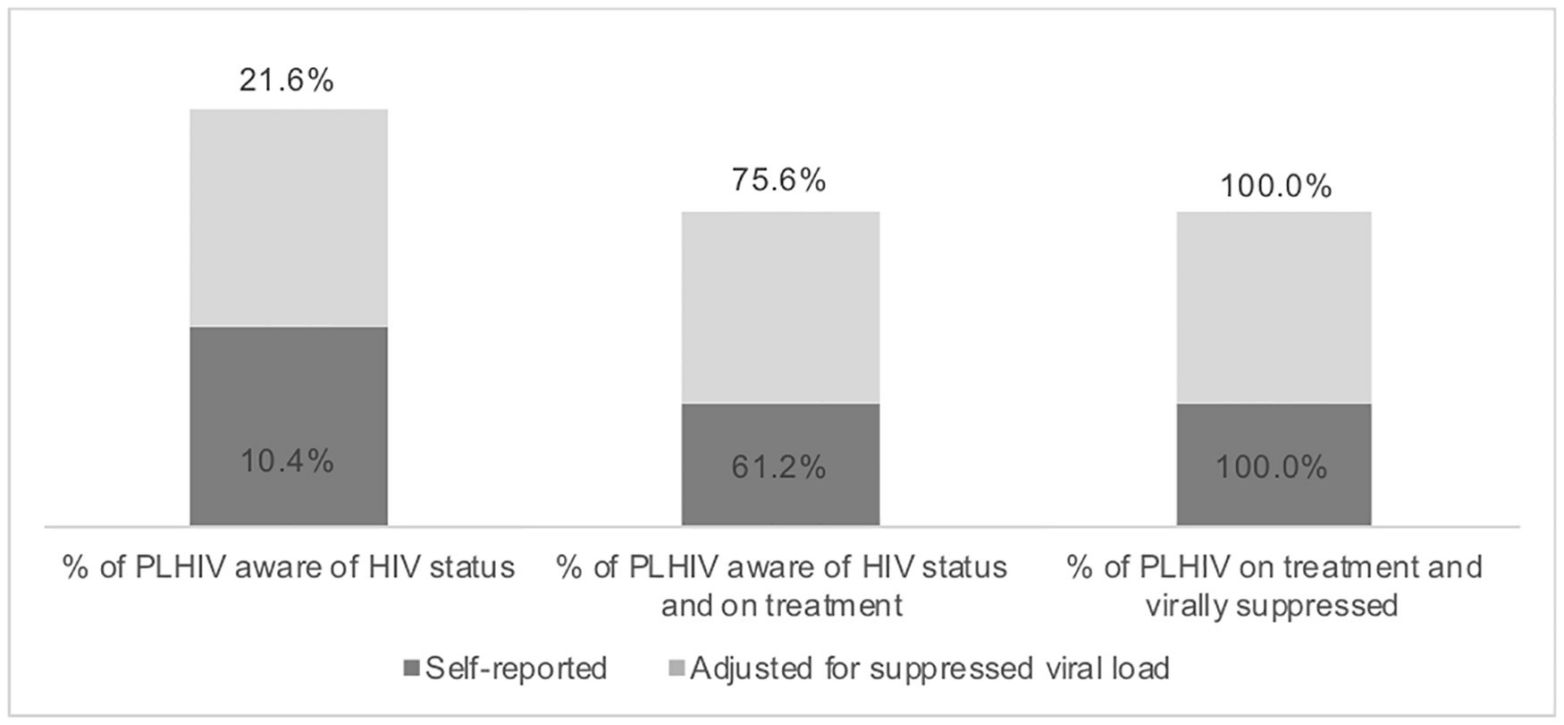
90-90-90 cascade among HIV-positive MSM and TGW in Bamako, Mali 2014–2015.

HIV prevalence was 13.7% (95% CI: 9.4–18.0) among MSM and TGW. Among HIV-positive MSM and TGW in Bamako, 10.4% (95% CI: 2.7–18.0) self-reported that they were were living with HIV (Fig 1). Of those aware of their HIV infection, 61.2% (95% CI: 10.2–100.0) were self-reported on ART, and of this group, 100% were virally suppressed (95% CI: 70.1–100.0). Among all HIV-infected MSM and TGW regardless of awareness of one’s status, 18.4% (95% CI: 8.1–28.7) were virally suppressed. Assuming that all people who are virally suppressed are aware of their HIV infection and on treatment, the cascade changes as follows (Fig 1): 21.6% (95% CI: 10.8–32.5) were aware of their HIV infection, of them 75.6% (95% CI: 51.7–99.6) were on treatment, and of them, 100% were virally suppressed (95% CI: 85.7–100.0). The geometric mean viral load was 19,356 copies/mL (95% CI: 8,070–46,428) and 9.9% (95% CI: 6.0–13.9) of all MSM/TGW had a viral load >1,500 copies/mL.

Looking at MSM and TGW separately, HIV prevalence was 9.5% (95% CI: 5.6–13.5) among MSM and 24.6% (95% CI: 13.9–35.3) among TGW. Among MSM, 14.8% (95% CI: 2.2–27.4) self-reported that they were living with HIV; this increases to 34.1% (95% CI: 15.6–52.5) if we assume that all MSM with suppressed viral load were aware of their infection. Among TGW, 5.9% (95% CI: 0.0–13.9) self-reported that they were living with HIV; this increases to 9.1% (95% CI: 0.0–18.3) if we assume that all TGW with suppressed viral load were aware of their infection.

Among MSM and TGW living with HIV, 41.5% were aged 18–24 years, 35.0% were 25–29 years, and 23.5% were ≥30 years, though there was no significant difference by awareness of HIV status or viral load suppression (Table 1). Whereas 23.1% of Malians were aware of their HIV infection, only 13.0% of non-Malians were aware of their HIV status. Awareness of HIV infection was higher among MSM than TGW (34.1% compared to 9.1%) (p = 0.0116). Awareness was 55.6% among MSM and TGW who screened positive for problematic consumption of alcohol and 17.6% among those screening negative (p = 0.0054).

**Table 1:** Characteristics of HIV-positive MSM and TGW in Bamako, Mali based on awareness of HIV-positive status and viral load suppression status, 2014–2015.

	HIV-positive (N = 79)	Aware (N = 27)	Unaware (N = 52)	*P-Value*	Virally Suppressed* (N = 23)	Not Virally Suppressed* (N = 51)	*P-Value*
	N (%; 95% CI)	N (%; 95% CI)	N (%; 95% CI)		N (%; 95% CI)	N (%; 95% CI)	
Age (years)				0.8754			0.3534
18–24	38 (41.5; 25.3–57.7)	10 (22.8; 6.0–39.6)	28 (77.2; 60.4–94.0)		6 (10.5; 0.1–20.9)	31 (89.5; 79.1–99.9)	
25–29	27 (35.0; 18.8–51.1)	9 (17.9; 1.6–34.3)	18 (82.1; 65.7–98.4)		9 (21.0; 0.7–41.2)	16 (79.0; 58.8–99.3)	
≥30	14 (23.5; 7.8–39.2)	8 (25.1; 0.0–51.0)	6 (74.9; 49.0–100.0)		8 (31.1; 0.0–69.6)	4 (68.9; 30.4–100.0)	
Highest level of education				0.5769			0.2526
Never attended school	7 (16.0; 1.4–30.7)	1 (9.3; 0.0–28.9)	6 (90.7; 71.1–100.0)		1 (9.9; 0.0–39.4)	5 (90.1; 60.6–100.0)	
Primary	23 (27.5; 12.3–42.7)	5 (15.8; 0.0–33.3)	18 (84.2; 66.7–100.0)		5 (16.7; 0.0–39.5)	17 (83.3; 63.5–100.0)	
Secondary	28 (32.2; 16.8–47.6)	11 (27.2; 5.8–48.6)	17 (72.8; 51.4–94.2)		8 (13.3; 0.0–26.8)	20 (86.7; 73.2–100.0)	
University	21 (24.3; 11.1–37.5)	10 (28.9; 5.8–52.0)	11 (71.1; 48.0–94.2)		9 (40.1; 7.2–73.0)	9 (59.9; 27.0–92.8)	
Marital status				0.9675			0.8509
Never married	73 (86.4; 71.8–100.0)	4 (21.0; 0.0–53.4)	2 (79.0; 46.6–100.0)		4 (21.0; 0.0–66.6)	2 (79.0; 33.4–100.0)	
Married, divorced, separated, or widowed	6 (13.6; 0.0–28.2)	23 (21.7; 10.3–33.2)	50 (78.3; 66.8–89.7)		19 (17.9; 7.2–28.7)	49 (82.1; 71.3–92.8)	
Money earned last month				0.5744			0.0492
< 50,000 CFA ($100 USD)	42 (60.0; 44.2–75.8)	12 (18.3; 4.7–31.8)	30 (81.7; 68.2–95.3)		9 (11.0; 1.5–20.4)	32 (89.0; 79.6–98.5)	
≥ 50,000 CFA ($100 USD)	36 (40.0; 24.2–55.8)	14 (24.2; 7.5–40.8)	22 (75.8; 59.2–92.5)		13 (29.4; 8.5–50.2)	19 (70.6; 49.8–91.5)	
Nationality				0.5676			0.6969
Malian	74 (85.8; 71.1–100.0)	26 (23.1; 11.4–34.7)	48 (76.9; 65.3–88.6)		22 (19.4; 8.5–30.4)	47 (80.6; 69.6–91.5)	
Other African nationalities	5 (14.2; 0.0–28.9)	1 (13.0; 0.0–40.1)	4 (87.0; 59.9–100.0)		1 (13.0; 0.0–55.0)	4 (87.0; 45.0–100.0)	
Gender Identity				0.0116			0.0153
Male	42 (50.2; 33.2–67.0)	20 (34.1; 15.6–52.5)	22 (65.9; 47.5–84.4)		17 (30.0; 11.5–48.6)	22 (70.0; 51.4–88.5)	
Transgender woman	37 (49.8; 33.0–66.7)	7 (9.1; 0.0–18.3)	30 (90.9; 81.7–100.0)		6 (7.2; 0.0–15.8)	29 (92.8; 84.2–100.0)	
Self-identifies as homosexual				0.1813			0.1641
Yes	36 (45.4; 28.4–62.4)	10 (14.1; 1.4–26.8)	26 (85.9; 73.2–98.6)		9 (11.6; 0.0–23.3)	26 (88.4; 76.7–100.0)	
No	43 (54.6; 37.6–71.6)	17 (27.9; 11.7–44.1)	26 (72.1; 55.9–88.3)		14 (25.3; 8.5–42.1)	25 (74.7; 57.9–91.5)	
Social cohesion				0.5062			0.0718
High	44 (47.1; 30.4–63.8)	17 (25.5; 9.5–41.5)	27 (74.5; 58.5–90.5)		16 (28.8; 9.9–47.7)	25 (71.2; 52.3–90.1)	
Low	35 (52.9; 36.2–69.6)	10 (18.2; 3.6–32.8)	25 (81.8; 67.2–96.4)		7 (10.3; 0.0–21.1)	26 (89.7; 78.9–100.0)	
Screened positive for depression				0.9368			0.5067
Yes	33 (45.0; 28.0–62.0)	13 (22.1; 5.4–38.8)	20 (77.9; 61.2–94.6)		12 (22.1; 4.1–40.2)	20 (77.9; 59.8–95.9)	
No	46 (55.0; 37.9–72.0)	14 (21.2; 6.9–35.5)	32 (78.8; 64.5–93.1)		11 (15.2; 2.8–27.5)	31 (84.8; 72.5–97.2)	
Problematic consumption of alcohol (AUDIT-C score ≥ 4)				0.0054			0.0077
Yes	15 (10.6; 3.8–17.4)	9 (55.6; 24.5–86.8)	6 (44.4; 13.2–75.5)		8 (48.2; 13.4–83.0)	7 (51.8; 17.0–86.6)	
No	64 (89.4; 82.6–96.2)	18 (17.6; 7.0–28.2)	46 (82.4; 71.8–93.0)		15 (14.4; 4.6–24.2)	44 (85.6; 75.8–95.4)	

*5 participants lacked viral load results due to insufficient specimen

Viral suppression among those on treatment was 10.5% among those ages 18–24 years, 21.0% among 25–29 year olds, and 31.1% among those 30 years and older (Table 1). Though not statistically significant, those with a university degree were most likely to be suppressed (40.1% compared to <20.0% for all other education levels). Eleven percent of those earning less than 50,000 CFA (approximately 100 USD) per month were virally suppressed versus 29.4% of those earning more at least that amount (p = 0.0492). While 30.0% of males were virally suppressed, 7.2% of TGW were virally suppressed (p = 0.0153). Viral suppression was 11.6% among those who self-identify as homosexual and 25.3% among those who do not. An estimated 9.5% of HIV-infected MSM or TGW who sell sex were virally suppressed.

Of those who used free condoms in the last six months, 29.5% were aware of their HIV infection compared to 6.5% of those who did not (p = 0.0126) (Table 2). Almost half (47.0%) of MSM/TGW were able to get condoms when they needed them and 88.5% used water-based lubricant in the last six months. Just over half (52.6%) of MSM/TGW had one male sex partner in the last six months compared to 22.0% who had three or more. Awareness of HIV status was higher for those with comprehensive knowledge of HIV (37.2%) compared to those without (7.6%, p = 0.0029). Among MSM and TGW who had unprotected anal intercourse with most recent partner at last sex, 27.6% were aware of their HIV status. Of those who had contact with an HIV outreach worker in the last year, 29.7% were aware of their status compared to 9.2% of those who had no contact with an outreach worker. Among MSM and TGW who did not use a condom at last sex, 21.3% were virally suppressed. Viral load suppression was higher among those with comprehensive knowledge of HIV (33.9%) compared to those without (5.7%, p = 0.0030).

**Table 2:** Comparison of sexual behaviors, HIV knowledge, and condom and lubricant use among HIV-positive MSM and TGW in Bamako, Mali based on awareness of HIV-positive status and viral load suppression status, 2014–2015.

	HIV-positive (N = 79)	Aware (N = 27)	Unaware (N = 52)	*P-Value*	Virally Suppressed* (N = 23)	Not Virally Suppressed* (N = 51)	*P-Value*
	N (%; 95% CI)	N (%; 95% CI)	N (%; 95% CI)		N (%; 95% CI)	N (%; 95% CI)	
**Sexual behaviors**							
Number of male sexual partners in past 6 months				0.0978			0.2173
1	33 (52.6; 35.8–69.3)	7 (10.8; 0.9–20.7)	26 (89.2; 79.3–99.1)		6 (10.5; 0.0–21.4)	24 (89.5; 78.6–100.0)	
2	18 (25.4; 10.1–40.7)	8 (32.5; 3.4–61.7)	10 (67.5; 38.3–96.6)		7 (24.4; 0.0–51.4)	10 (75.6; 48.6–100.0)	
3+	28 (22.0; 10.8–33.2)	12 (34.9; 12.0–57.9)	16 (65.1; 42.1–88.0)		10 (30.7; 7.8–53.6)	17 (69.3; 46.4–92.2)	
Any female sexual partners—last 6 months				0.6343			0.9726
Yes	31 (45.1; 26.8–63.5)	12 (20.1; 3.5–36.6)	19 (79.9; 63.4–96.5)		11 (19.9; 1.9–37.9)	19 (80.1; 62.1–98.1)	
No	38 (54.9; 36.5–73.2)	13 (25.6; 8.7–42.5)	25 (74.4; 57.5–91.3)		7 (20.3; 2.7–38.0)	22 (79.7; 62.0–97.3)	
Had concurrent sexual activity with last male partner				0.4447			0.7668
Yes	48 (66.7; 48.6–84.8)	18 (22.1; 7.5–36.7)	30 (77.9; 63.3–92.5)		15 (15.3; 3.7–26.9)	30 (84.7; 73.1–96.3)	
No	18 (33.3; 15.2–51.4)	4 (13.4; 0.0–29.2)	14 (86.6; 70.8–100.0)		3 (12.2; 0.0–30.1)	14 (87.8; 69.9–100.0)	
Has received money, goods, or services in exchange for sex—last 6 months				0.5637			0.2853
Yes	13 (11.7; 2.6–20.9)	5 (30.4; 0.0–67.1)	8 (69.6; 32.9–100.0)		4 (9.5; 0.0–23.3)	9 (90.5; 76.7–100.0)	
No	66 (88.3; 79.1–97.4)	22 (20.5; 9.4–31.5)	44 (79.5; 68.5–90.6)		19 (19.8; 8.0–31.6)	42 (80.2; 68.4–92.0)	
Reported any STI symptom, last 12 months				0.1568			0.8855
Yes	22 (26.2; 11.5–40.9)	11 (35.5; 7.9–63.2)	11 (64.5; 36.8–92.1)		8 (19.6; 0.0–39.4)	13 (80.4; 60.6–100.0)	
No	57 (73.8; 59.1–88.5)	16 (16.7; 5.7–27.6)	41 (83.3; 72.4–94.3)		15 (18.0; 5.5–30.4)	38 (82.0; 69.6–94.5)	
**Condom and lubricant use**							
Did not use a condom during last anal sex with most recent partner				0.1540			0.4885
Yes	62 (62.7; 45.3–80.2)	24 (27.6; 13.2–42.0)	38 (72.4; 58.0–86.8)		20 (21.3; 8.6–34.0)	39 (78.7; 66.0–91.4)	
No	17 (37.3; 19.8–54.7)	3 (11.6; 0.0–25.9)	14 (88.4; 74.1–100.0)		3 (13.4; 0.0–32.0)	12 (86.6; 68.0–100.0)	
Had a condom break during anal sex with a man—last 6 months				0.1365			<0.0001
Yes	18 (33.6; 15.9–51.3)	3 (9.2; 0.0–24.2)	15 (90.8; 75.8–100.0)		2 (2.2; 0.0–6.1)	15 (97.8; 93.9–100.0)	
No	61 (66.4; 48.7–84.1)	24 (27.9; 14.1–41.7)	37 (72.1; 58.3–85.9)		21 (26.7; 12.2–41.2)	36 (73.3; 58.8–87.8)	
Used free condoms—last 6 months				0.0126			0.1426
Yes	57 (66.0; 49.5–82.4)	23 (29.5; 13.8–45.1)	34 (70.5; 54.9–86.2)		19 (22.5; 8.7–36.2)	36 (77.5; 63.8–91.3)	
No	22 (34.0; 17.6–50.5)	4 (6.5; 0.0–14.6)	18 (93.5; 85.4–100.0)		4 (8.5; 0.0–20.3)	15 (91.5; 79.7–100.0)	
Able to get condoms when need one—last 6 months				0.3765			0.0588
Yes	27 (47.0; 29.8–64.3)	6 (16.6; 0.8–32.3)	21 (83.4; 67.7–99.2)		19 (27.0; 11.4–42.6)	29 (73.0; 57.4–88.6)	
No	52 (53.0; 35.7–70.2)	21 (26.1; 12.1–40.2)	31 (73.9; 59.8–87.9)		4 (9.4; 0.0–20.7)	22 (90.6; 79.3–100.0)	
Used water-based lubricant during anal sex- last 6 months				0.7306			0.8976
Yes	72 (88.5; 79.2–97.8)	26 (22.3; 10.6–34.0)	46 (77.7; 66.0–89.4)		22 (18.2; 7.6–28.7)	47 (81.8; 71.3–92.4)	
No	7 (11.5; 2.2–20.8)	1 (16.2; 0.0–46.2)	6 (83.8; 53.8–100.0)		1 (20.6; 0.0–58.6)	4 (79.4; 41.3–100.0)	
**Disclosure and Discrimination**							
Has disclosed their sexual orientation to someone else other than their sexual partner				0.5974			0.6792
Yes	54 (78.4; 62.3–94.5)	15 (18.7; 6.5–31.0)	39 (81.3; 69.0–93.5)		13 (16.8; 4.7–29.0)	36 (83.2; 71.0–95.3)	
No	13 (21.6; 5.5–37.7)	6 (26.6; 0.0–56.0)	7 (73.4; 44.0–100.0)		5 (22.6; 0.0–53.0)	8 (77.4; 47.0–100.0)	
Been blackmailed for having sex with men							
Yes	19 (21.0; 5.8–36.2)	9 (15.6; 0.0–33.0)	10 (85.4; 67.0–100.0)		8 (14.3; 0.0–33.4)	10 (85.7; 77.1–100.0)	
No	60 (79.0; 63.8–94.2)	18 (23.2; 10.5–35.9)	42 (76.8; 64.1–89.5)		15 (19.4; 7.4–31.4)	41 (80.6; 68.6–92.6)	
Suffered harassment or abuse because they had sex with men				0.6192			0.8221
Yes	28 (26.2; 11.2–41.2)	13 (26.2; 3.6–48.8)	15 (73.8; 51.2–96.4)		10 (20.6; 0.0–44.7)	15 (79.4; 55.3–100.0)	
No	51 (73.8; 58.8–88.8)	14 (20.0; 7.6–32.4)	37 (80.0; 67.6–92.4)		13 (17.8; 6.2–29.4)	36 (82.2; 70.6–93.8)	
**HIV Knowledge**							
Comprehensive HIV knowledge				0.0029			0.0030
Yes	45 (47.4; 30.8–63.9)	21 (37.2; 19.1–55.3)	24 (62.8; 44.7–80.9)		18 (33.9; 16.0–51.8)	25 (66.1; 48.2–84.0)	
No	34 (52.6; 36.1–69.2)	6 (7.6; 0.0–16.6)	28 (92.4; 83.4–100.0)		5 (5.7; 0.0–14.1)	26 (94.3; 85.9–100.0)	
Had contact with HIV outreach—last year				0.0912			0.2557
Yes	56 (79.4; 63.2–95.6)	22 (29.7; 14.0–45.4)	34 (70.3; 54.6–86.0)		18 (23.6; 9.3–37.9)	36 (76.4; 62.1–90.7)	
No	14 (20.6; 4.4–36.8)	4 (9.2; 0.0–23.0)	10 (90.8; 77.0–100.0)		4 (10.1; 0.0–25.5)	9 (89.9; 74.5–100.0)	

*5 participants lacked viral load results due to insufficient specimen

In multivariate analysis, not using free condoms in the last six months was associated with being unaware of HIV infection (aOR: 5.7, 95% CI: 1.4–28.8) (Table 3). The relative odds of being unaware of being HIV infected were 6.5 times higher (95% CI: 1.4–29.2) for MSM and TGW who did not have comprehensive knowledge of HIV than for those who did. While not significant, transgender women were more likely to be unaware than people identifying as male (aOR: 3.8, 95% CI: 0.9–16.7). Among all MSM and TGW living with HIV in Bamako, having unsuppressed viral load was associated with identifying as a transgender woman (aOR: 4.8, 95% CI: 1.1–20.2) and not having comprehensive knowledge of HIV (aOR: 6.5, 95% CI: 1.1–39.7) (Table 4).

**Table 3:** Factors associated with being unaware of having HIV among MSM and TGW in Bamako, Mali 2014–2015.

	Bivariate analysis		Multivariate analysis	
	OR (95% CI)	p-value	aOR (95% CI)	p-value
Gender Identity		0.0191		0.0754
Male	Ref		Ref	
Transgender woman	5.2 (1.3–20.4)		3.8 (0.9–16.7)	
Problematic consumption of alcohol (AUDIT-C score greater than or equal to 4)		0.0165		
Yes	Ref			
No	5.9 (1.4–24.9)			
Used free condoms—last 6 months		0.0208		0.0345
Yes	Ref		Ref	
No	6.0 (1.3–27.7)		5.7 (1.4–28.8)	
Comprehensive HIV knowledge		0.0093		0.0154
Yes	Ref		Ref	
No	7.2 (1.6–31.7)		6.5 (1.4–29.2)	

**Table 4:** Factors associated with not having suppressed viral load among MSM and TGW in Bamako, Mali 2014–2015.

	Bivariate analysis		Multivariate analysis	
	aOR (95% CI)	p-value	aOR (95% CI)	p-value
Money earned last month		0.0771		
< 50,000 CFA ($100 USD)	3.4 (0.9–13.0)		---	
≥ 50,000 CFA ($100 USD)	Ref		---	
Gender Identity		0.0253		0.0345
Male	Ref		Ref	
Transgender woman	5.5 (1.2–24.6)		4.8 (1.1–20.2)	
Social cohesion		0.0853		0.1106
High	Ref		Ref	
Low	3.5 (0.8–14.7)		3.3 (0.8–14.8)	
Problematic consumption of alcohol (AUDIT-C score greater than or equal to 4)		0.0229		
Yes	Ref			
No	5.5 (1.2–24.2)			
Had a condom break during anal sex with a man—last 6 months		0.0027		
Yes	16.2 (2.6–99.7)			
No	Ref			
Able to get condoms when need one—last 6 months		0.0913		
Yes	3.6 (0.8–15.5)			
No	Ref			
Comprehensive HIV knowledge		0.0131		0.0409
Yes	Ref		Ref	
No	8.5 (1.6–46.5)		6.5 (1.1–39.7)	

Of the survey participants who tested for HIV, 79 tested positive. All positives were tested by the LAg assay. Eleven specimens were LAg-positive and all but 2 with insufficient specimen were tested for viral load. Of the 9 LAg-positive specimens tested for viral load, 5 had suppressed viral load and were classified as non-recent while 4 were not suppressed and were classified as recent. This yields a proportion of recent infections of 5.1% (95% CI: 2.0–12.6) among survey participants. The weighted percentage testing recent was 2.0% (95% CI: 0.5–8.5).

## Discussion

UNAIDS 90-90-90 targets offer hope for a way to control the HIV epidemic. Unfortunately, like in many other countries, achievements among MSM and TGW in Bamako are far from these targets.[4, 23, 24] Even with the most liberal of assumptions that all people who are virally suppressed are also aware of their HIV status and on treatment, less than one-quarter of MSM and TGW living with HIV in Bamako are aware of their HIV status. Though self-reported awareness is higher among MSM than TGW, when we assume that those with viral suppression are aware of their infection, there is no significant difference between the two populations. Nonetheless, as elsewhere, HIV prevalence was higher among TGW than MSM.[25]

As reported elsewhere, 45.2% of MSM and TGW in Bamako used free condoms in the last six months and 27.8% simultaneously experienced times when they were unable to obtain condoms.[20] Here we found that not using free condoms in the last six months was associated with a greater odds of having undiagnosed HIV infection (aOR: 5.7, 95% CI: 1.4–28.8). This may be because people who have been diagnosed have more opportunities to obtain free condoms as a result of visits to care or treatment clinic visits or peer support groups. They may also be more inclined to use condoms to protect others, whereas those who are undiagnosed may not believe themselves to be at risk of HIV.[20] Our finding that comprehensive knowledge of HIV, which only 47.4% of HIV-infected and 56.7% of all MSM and TGW in Bamako possess, is associated with both being aware of infection and viral suppression suggests a continued role for basic HIV education tied with HIV testing.[20] It is likely that TGW were more likely to have unsuppressed viral load than MSM because they were also more likely to be unware that they had HIV and therefore could not be on ART.

Reaching 90-90-90 targets for MSM and TGW in Bamako will likely be harder than for the general population because of the limited services that target them and the additional structural barriers these populations face to access HIV services.[20, 26–29] Despite these challenges, it is worth noting that among MSM and TGW on treatment, all were virally suppressed, indicating very good adherence. Nonetheless, as so few MSM and TGW have been diagnosed with HIV and so few are on treatment, treatment will only go so far in slowing the spread of HIV. Other prevention strategies are needed. Furthermore, though everyone on treatment was virally suppressed, viral load monitoring among those on ART remains critical as it may be harder to maintain such high suppression as more people are started on treatment.

A chronic infection, HIV is associated with increased age in Bamako; nevertheless, 76.5% of MSM and TGW living with HIV are less than 30 years old.[20] Low levels of awareness, treatment uptake, and viral suppression, combined with a young population living with HIV and low condom use among MSM and TGW, suggests substantial opportunity for HIV to spread among these populations in Bamako.[21] Increased resources for HIV testing and improved testing strategies are urgently needed to control the HIV epidemic among MSM and TGW in Bamako. While countries close to reaching the first 90 among the general population are now targeting specific sub-populations for HIV testing, a concerted testing campaign is needed for all MSM and TGW in Bamako. Additionally, in this setting where awareness of HIV status is low and most (62.7%) MSM and TGW living with HIV did not use a condom at last anal sex, condom and water-based lubricant promotion and distribution can still play a key role in HIV prevention efforts. Water-based lubricant use in the last six months was higher than expected; however, we do not know how frequently this was used compared to no lubricant or an oil-based lubricant.[11, 30] A qualitative survey could uncover more about this and why one-third of MSM and TGW had a condom break during anal sex in the last six months.

Like most surveys of key populations, our sample size prevented the estimation of incidence using laboratory-based methods. We were able to estimate the proportion of infections that are recent, however. In our sample, 5.1% of people living with HIV had a recent infection, similar to that found in the general population 2012 Kenya AIDS Indicator Survey (5.9%) which had an incident rate of 0.5% (95% CI: 0.2–0.9) and population viral suppression twice as high at 38.8% than in our survey.[31, 32] The proportion of infections that were recent is higher, however, than that found in the general population 2015–2016 Zimbabwe Population-based HIV Impact Assessment (1.0%) with a similar incident rate to Kenya (0.5%, 95% CI: 0.3–0.6) but higher population viral suppression (55.7%).[33, 34]

While our sample size was larger than that of most BBS conducted through September 2013, our survey was statistically powered for HIV prevalence rather than viral load suppression, hampering our analysis, including our ability to describe the 90-90-90 cascades for MSM and TGW separately.[7] In order to detect associations with each step of the treatment cascade and changes within steps over time, surveys should be powered for viral load suppression. This will require additional financial resources and may necessitate the aggregating of data across survey sites. Social desirability bias may have resulted in inaccurate reporting of HIV testing and treatment history as seen in the sensitivity analysis (Fig 1). Shame and stigma may play a role in this underreporting. The use of audio computer-assisted self-interviews could help reduce social desirability bias in future surveys.[35, 36] Testing for the presence of antiretroviral medications could facilitate estimation of the true population proportion of people who are aware that they are living with HIV and on antiretroviral therapy.[37] Our survey would have been strengthened had we been able to conduct separate surveys on MSM and TGW. Though these are two different populations with different risks and service needs, resource limitations and the inability to achieve a sufficient sample size in a survey of only TGW led us to include both populations in a single sample rather than restricting the sample to only MSM. Finally, as this was a cross-sectional survey, we are only able to assess associations and not causality. It may be, for instance, that people with comprehensive knowledge of HIV were more likely to be tested for HIV. It could also be that people received information about HIV upon diagnosis and consequently developed comprehensive knowledge.

There is an urgent need for increased HIV testing among MSM and TGW in Bamako and improved linkages from testing to treatment initiation. Despite slow progress towards achievement of the first two 90s, there is hope in the impressive viral suppression among those on treatment, indicating that adherence and retention are good and suggesting that drug resistance may not be a concern for the treatment of MSM and TGW.

## Supporting information

S1 Appendix Teriya English questionnaire. (DOC)

S2 Appendix Teriya French questionnaire. (PDF)
