# Supplementary material for: An urgent need for HIV testing among men who have sex with men and transgender women in Bamako, Mali: Low awareness of HIV infection and viral suppression among those living with HIV
Source: PLoS One. 2018 Nov 12;13(11):e0207363. doi: 10.1371/journal.pone.0207363 (PMC6231666; doi:10.1371/journal.pone.0207363)
Supplement: S1 Appendix — (DOC) [file pone.0207363.s001.doc]

**Appendix 5.** Questionnaire 1st visit

**SURVEY QUESTIONNAIRE (1st VISIT)**

TERIYA STUDY

| **Before beginning the interview, please make sure that the screening form has been completed and recorded on the checklist and that informed consent has been obtained and recorded on the checklist.** |
| --- |

| **Introduction:** « ***Hello, my name is… I will help you to do the interview. You can refuse to answer any question and you can stop the interview at any time. Answering all the questions will help make this survey a success. If you do not know the exact answer to a question try to give your best guess. For most questions, choose the one answer that fits best. We will tell you if a question allows for more than one answer. If you need help, just ask. We keep all your answers confidential. We will not ask for your name or other information that tells who you are»*** |
| --- |

| ***Questionnaire parameters*** | | | |
| --- | --- | --- | --- |
| **T01** | Interviewer Code (initials)  *Please enter only your initials (two letters).* |  | |
| **T02a** | Study site | Site A  Site B | 1  2 |
| **T02b** | Study ID  *Each study participant is assigned a four-digit sequential study ID number: XZZZ, where ""X"" designates the study site (A or B), and ""ZZZ"" is the running number starting with participant 001.*  *The study ID should only contain the letter indicating the study site and the three digits assigned to the participant.* |  | |
| **start_time_date** | Start time and date of interview *(automatic)* |  |  |
| **end_time_date** | End time and date of interview *(automatic)* |  |  |

| **No.** | **QUESTIONS** | **CODING CATEGORIES** | |  | **SKIPS** | |
| --- | --- | --- | --- | --- | --- | --- |
| 1. ***RECRUITER INFORMATION***   **I’m going to start by asking you about the person who gave you the coupon and about other men who have sex with men you know in Bamako. Please remember that your answers will be kept private.** | | | | | | |
|  | Which of the following describes how you know the person who gave you this coupon? | A relative or family member  A sex partner  A friend  An acquaintance  A stranger  Other  Refuse to answer | | 1  2  3  4  5  777  999 | If 1, 3, 4, 5, 777, or 999 go to Q3. | |
|  | What kind of a sex partner is this person?  *The interviewer can clarify the types of sex partners using the handout available during the interview.* | Main partner  Casual partner  Commercial- I paid him  Commercial- He paid me  Don’t know  Refuse to answer | | 1  2  3  4  888  999 |  | |
|  | Do you know this person for some days, or some months, or for years? | Days  Months  Years  Don’t know  Refuse to answer | | 1  2  3  888  999 |  | |
|  | How many times have you seen this person in the last 30 days? | Number: |__|__]  Range: 0-30  Don’t know  Refuse to answer | | 888  999 |  | |
|  | Was your coupon exchanged for something? | No, nothing  Money or goods  Sexual favors  Other  Refuse to answer | | 1  2  3  777  999 |  | |
|  | What is the main reason you joined this study?  DO NOT READ ANSWERS  SELECT ONLY ONE | Interested in the study  Want to know my HIV status  Want the incentive  Was forced to come  Want to help the community  Other  Refuse to answer | | 1  2  3  4  5  777  999 |  | |
| 1. ***BACKGROUND CHARACTERISTICS***   **Now I am going to ask you some background questions about yourself. Remember that all your answers are confidential.** | | | | | | |
|  | How old are you? | Age (years)  Refuse to answer | | [_I_]  999 |  | |
|  | What is your nationality?  DO NOT READ ANSWERS  SELECT ONLY ONE | Malien  Ivorien  Burkinabe  Senegalais  Ghanaian  Guinean  Liberian  Togolais  Beninois  Nigerian  Other African  Other Non-African  Refuse to answer | | 1  2  3  4  5  6  7  8  9  10  11  12  999 |  | |
|  | What countries or places in Mali have you visited in the past 6 months?  DO NOT READ ANSWERS  SELECT ALL THAT APPLY | Other city in Mali (besides Bamako)  Cote d’Ivoire  Burkina Faso  Ghana  Guinée  Libéria  Togo  Bénin  Nigéria  Other African countries  Other Non-African countries  Refuse to answer | | 1  2  3  4  5  6  7  8  9  10  11  999 |  | |
|  | In which commune of Bamako do you live?  DO NOT READ ANSWERS SELECT ONLY ONE | Commune 1  Commune 2  Commune 3  Commune 4  Commune 5  Commune 6  Suburbs  Refuse to answer | | 1  2  3  4  5  6  7  999 | If 1,2,3,4,5,6 or 999 go to Q12 | |
|  | In which suburb do you live?  DO NOT READ ANSWERS SELECT ONLY ONE | Kati  Kalabankoro  Senou  Moribabougou  Sangarebougou  Titibougou  Baguineda  Safo  Sirakoro Megatana  Niamana  Samaya  Dialakorodji  Dialakoroba  Sanakoroba  Other  Refuse to answer | | 1  2  3  4  5  6  7  8  9 10  11  12  13  14  777  999 |  | |
|  | Can you read and write? | Cannot read and write  Can read only  Can read and write  Don’t know  Refuse to answer | | 1  2  3  888  999 |  | |
|  | What is the highest level of studies you have achieved? | Never attended  Bambara alphabetization  Primary  Secondary  University  Post-University  Refuse to answer | | 1  2  3  4  5  6  999 | If 1 go to Q15. | |
|  | What is the last year or class that you have attended? | Year/Class  Don’t know  Refuse to answer | | [_I_]  888  999 |  | |
|  | Have you ever attended a vocational training school? | Yes  No  Refuse to answer | | 1  2  999 |  | |
|  | What is your main occupation?  DO NOT READ ANSWERS  SELECT ONLY ONE | Have no work  Student  Hawker, street vendor, casual laborer, etc.  Mechanic, factory worker, laborer  Professional/teacher/banker/accountant  Hairdresser  Waiter/bar manager/hotel etc.  Uniformed services  Sex worker  Artisan/musician/dancer/performer, etc.  Taxi/bus driver  Farmer  Security  Civil servant  Other  Refuse to answer | | 1  2  3  4  5  6  7  8  9  10  11  12  13  14  777  999 |  | |
|  | How much money did you earn last month?  SELECT ONLY ONE | < 25 000 CFA  25 000 – 49 999 CFA  50 000 – 149 999 CFA  150 000 – 299 999 CFA  > 300 000 CFA  Don’t know  Refuse to answer | | 1  2  3  4  5  888  999 |  | |
|  | What religion are you?  DO NOT READ ANSWERS  SELECT ONLY ONE | Muslim  Christian  Animist  Other religion  No religion  Refuse to answer | | 1  2  3  4  5  999 |  | |
|  | What is your current marital status?  SELECT ONLY ONE | Never married  Married (to one woman)  Married (to more than one woman)  Divorced, separated, or widowed  Refuse to answer | | 1  2  3  4  999 |  | |
|  | What kind of place do you live in?  READ ANSWERS ALOUD  SELECT ONLY ONE | House  Apartment  Dormitory  Community center  Street/homeless  Other  Refuse to answer | | 1  2  3  4  5  777 999 |  | |
|  | Do you have a regular place to sleep at night? | Yes  No  Don’t know  Refuse to answer | | 1  2  888  999 |  | |
|  | Over the last 2 weeks, how often have you had little interest or pleasure in doing things? | Not at all  Several days  More than half the days  Nearly every day  Refuse to answer | | 1  2  3  4  999 |  | |
|  | Over the last 2 weeks, how often have you been feeling down, depressed or hopeless? | Not at all  Several days  More than half the days  Nearly every day  Refuse to answer | | 1  2  3  4  999 |  | |
|  | How often do you have a drink containing alcohol? | Never  Monthly or less  2-3 times a month  2-3 times a week  4 or more times a week  Refuse to answer | | 1  2  3  4  5  999 | If 1, go to Q27. | |
|  | How many standard drinks containing alcohol do you have on a typical day?  *By standard drink we mean one bottle of beer (350ml), one glass of wine (150ml) or 45ml of liquor.* | 1 to 2  3 to 4  5 to 6  7 to 9  ≥10  Refuse to answer | | 1  2  3  4  5  999 |  | |
|  | How often do you have six or more drinks on one occasion? | Never  Less than monthly  Monthly  Weekly  Daily or almost daily  Refuse to answer | | 1  2  3  4  5  999 |  | |
|  | Some people take non-injectable drugs or medicine for pleasure or for reasons not recommended by a doctor. Have you taken drugs for these reasons in the past 6 months? | Yes  No  Refuse to answer | | 1  2  999 | If 2 or 999, go to Q29. | |
|  | In the last six months have you used:  *PROBE ALL* | Yes no d/k nr  Glue (sniffing) 1 2 888 999  Tobacco 1 2 888 999  Petrol (sniffing) 1 2 888 999  Marijuana (hashish) 1 2 888 999  Heroine (nono) 1 2 888 999  Cocaine 1 2 888 999  Amphetamine 1 2 888 999  Medicine 1 2 888 999  Other 1 2 888 999 | |  |  | |
|  | Some people inject drugs with a syringe for pleasure or for reasons not recommended by a doctor. Have you ever in your life injected any drugs other than those prescribed for you?  *By injecting, I mean anytime you have used any illegal or illicit drugs with a needle, either by injecting into the vein, injecting under the skin, or injecting into your muscle.* | Yes  No  Refuse to answer | | 1  2  999 | If 2 or 999, go to Q31. | |
|  | Have you ever shared a needle or syringe with someone else in the past 6 months? | Yes  No  Refuse to answer | | 1  2  999 |  | |
| 1. ***NETWORK SIZE***   **Now I’m going to ask you a few questions about how many people you know.** | | | | | | |
|  | How many men do you know who have sex with other men?  GET THE BEST ESTIMATE FROM PARTICIPANT | XXX  Number [__|__|__]  Refuse to answer | | 999 | If the participant responds with '0,' probe with the following question: "What about the person who gave you a coupon?"  If 0 or 999, go to Q37 | |
|  | Of these XXX men, how many live or work in Bamako and suburbs?  GET THE BEST ESTIMATE FROM PARTICIPANT | YYY  Number [__|__|__]  Max: XXX  Refuse to answer | | 999 | If 0 or 999, go to Q37 | |
|  | How many of these YYY men are 18 years or older?  GET THE BEST ESTIMATE FROM PARTICIPANT | ZZZ  Number [__|__|__]  Max: YYY  Refuse to answer | | 999 | If 0 or 999, go to Q37 | |
|  | How many of these ZZZ men have you seen in the past 30 days?  GET THE BEST ESTIMATE FROM PARTICIPANT | TTT  Number [__|__|__]  Max: ZZZ  Refuse to answer | | 999 | If 0 or 999, go to Q37 | |
|  | Of these TTT men, how many would you consider inviting to participate in this survey?  GET THE BEST ESTIMATE FROM PARTICIPANT | Number [__|__|__]  Min:000  Max: TTT  Refuse to answer | | 999 | If 0 or 999, go to Q37 | |
|  | For how many of the TTT men you saw in the last 30 days do you have their phone numbers in your cell phone?  GET THE BEST ESTIMATE FROM PARTICIPANT | Number [__|__|__]  Max: TTT  Refuse to answer | | 999 | If 0 or 999, go to Q37 | |
|  | Without counting the person who gave you this coupon, how many other MSM do you know that have already participated in this survey? | Number [__|__|__]  Refuse to answer | | 999 |  | |
| 1. ***SEXUAL IDENTITY AND HISTORY***   **The next few questions are about your gender identity and sexual orientation. All of your answers are confidential.**  **By homosexual/gay we mean that you identify as someone who is sexually attracted to someone of the same sex. By bisexual we mean someone who identifies as being attracted to both men and women. By heterosexual we mean that you identify as someone who is attracted to the opposite sex***.* | | | | | | |
|  | Do you mostly identify as male, female, transgender or something else? | Male  Female  Transgender  Something else/other  Refuse to answer | | 1  2  3  4  999 |  | |
|  | Would you describe yourself as:  READ ANSWERS ALOUD  SELECT ONLY ONE | Gay/Homosexual  Bisexual  Straight/heterosexual  Other  Don’t know  Refuse to answer | | 1  2  3  777  888  999 |  | |
|  | What sex would you say you are most sexually attracted to?  DO NOT READ ANSWERS  SELECT ONLY ONE | Only or mostly male  Equally male and female  Only or mostly female  Don’t know  Refuse to answer | | 1  2  3  888  999 |  | |
|  | Other than your male sex partners, who have you told that you are attracted to males?  CHECK ALL THAT APPLY | No one  Family members  Spouse or partner  Friends who are not gay, lesbian or bisexual  Friends who are gay, lesbian or bisexual  Health care provider  Other  Don’t know  Refuse to answer | | 1  2  3  4  5  6  777  888  999 |  | |
|  | About your family: Would you say that your family accepts that you are attracted to men, doesn’t know about it, or rejects you for it? | Accepts me  Doesn’t know  Rejects me  Refuse to answer | | 1  2  3  999 | If Q38≠3 (not trans), go to Q51. | |
|  | What was your physical sex assigned at birth? | Male  Female  Intersex/Ambiguous  Refuse to answer | | 1  2  3  999 | ONLY FOR PEOPLE WHO IDENTIFY AS TRANS (Q38=3) | |
|  | [In the past 6 months, have you ever lived as a woman? By living as a woman, I mean dressing and presenting yourself as a woman.](javascript:__doPostBack('ctl00$ContentPlaceHolder1$dgdQuestion$ctl06$ctl01','')) | Yes  No  Refuse to answer | | 1  2  999 | ONLY FOR PEOPLE WHO IDENTIFY AS TRANS (Q38=3) | |
|  | Which of the following people have you told about your transgender identity?  CHECK ALL THAT APPLY | Transgender friends  Gay/Lesbian friends who are not transgender  Heterosexual friends who are not transgender  Family  Spouse  Healthcare provider  Other  Don’t know  Refuse to answer | | 1  2  3  4  5  6  777  888  999 | ONLY FOR PEOPLE WHO IDENTIFY AS TRANS (Q38=3) | |
|  | Have you ever used hormones to change your body? | Yes  No  Refuse to answer | | 1  2  999 | ONLY FOR PEOPLE WHO IDENTIFY AS TRANS (Q38=3)  If 2 or 999, go to Q48. | |
|  | How often did you get hormone injections in the past 6 months? | Never  At least once a day  Once a week  Once a month  Refuse to answer | | 1  2  3  4  999 | ONLY FOR PEOPLE WHO IDENTIFY AS TRANS (Q38=3) | |
|  | Other than hormones, have you had a gender enhancement/or transition procedure? | Yes  No  Refuse to answer | | 1  2  999 | ONLY FOR PEOPLE WHO IDENTIFY AS TRANS (Q38=3) | |
|  | [Think about the last time you had sex with a male partner. At that time was this partner aware you were transgender?](javascript:__doPostBack('ctl00$ContentPlaceHolder1$dgdQuestion$ctl21$ctl01','')) | Yes  No  Don’t know  Refuse to answer | | 1  2  888  999 | ONLY FOR PEOPLE WHO IDENTIFY AS TRANS (Q38=3) | |
|  | Did you tell him that you were transgender? | Yes  No  Refuse to answer | | 1  2  999 | ONLY FOR PEOPLE WHO IDENTIFY AS TRANS (Q38=3) | |
| ***Sexual history***  **The next few questions are about your lifetime sexual history. This includes vaginal and anal sex. With vaginal sex we mean a penis enters a vagina. With anal sex we mean a penis enters a person’s anus (butt).** | | | | | | |
|  | Have you ever had oral, vaginal or anal sex with a woman? | Yes  No  Refuse to answer | | 1  2  999 | If 2 or 999 go to Q53. | |
|  | How old were you when you first had oral, vaginal or anal sex with a woman? | Age (years)  Max: Q7  Don’t know  Refuse to answer | | [_I_]  888  999 |  | |
|  | Have you ever had oral sex with a man? | Yes  No  Refuse to answer | | 1  2  999 |  | |
|  | Have you ever had anal sex with a man? | Yes  No  Refuse to answer | | 1  2  999 | If D53=2 and D54=2, recheck eligibility and stop the interview if ineligible. You must enter 'Refuse to respond' for Questions D55-M236. Then complete Questions T03-T05 before dismissing the participant. | |
|  | Please think back to the first time you had sex with a man. How old were you when you first had sex with a man? | Age (years)  Max: Q7  Don’t know  Refuse to answer | | [_I_]  888  999 |  | |
|  | Approximately how old was your partner?  *Please give your best guess* | 10-14 years  15-19 years  20-24 years  25-29 years  30-34 years  35-39 years  40 years or older  Don’t know  Refuse to answer | | 1  2  3  4  5  6  7  888999 |  | |
|  | Did he pay you or give you something in exchange for sex? | Yes  No  Don’t know  Refuse to answer | | 1  2  888  999 |  | |
|  | Did you pay or give him something to have sex? | Yes  No  Don’t know  Refuse to answer | | 1  2  888  999 |  | |
|  | How would you describe the way you knew him?  DO NOT READ ANSWERS ALOUD  SELECT ONLY ONE | Boyfriend/partner  Friend/acquaintance/coworker  Relative  Stranger  ‘Authority figure’ (government official religious leader, teacher, employer, military, police, prison guard)  Other  Don’t know  Refuse to answer | | 1  2  3  4  5  777  888  999 |  | |
|  | What kind of “authority” was he? | Professor/teacher  Religious leader  Employer  Military man/police officer  Prison guard  Other  Don’t know  Refuse to answer | | 1  2  3  4  5  777  888  999 | Only if Q59=5 | |
|  | Have you *ever* lived with a *male* sex partner? | Yes  No  Refuse to answer | | 1  2  999 |  | |
|  | Other than your male sex partners, have you told anyone about having sex with men? | Yes  No  Don’t know  Refuse to answer | | 1  2  888  999 | If 2, go to Q64. | |
|  | Who have you told about having sex with men?  PROBE ALL  CHECK ALL THAT APPLY. | Y N NR  MSM/Lesbian/Bi/Trans friends 1 2 999  Other friends 1 2 999  Spouse 1 2 999  Other family 1 2 999  Health care worker 1 2 999  Other 1 2 999 | |  |  | |
|  | Have you ever given a man something to have sex? By something I mean money, goods or services. | Yes  No  Don’t know  Refuse to answer | | 1  2  888  999 |  | |
|  | On which of these websites do you have an account/profile?  PROBE ALL | Yes No NR  gayromeo.com 1 2 999  draguenet.net 1 2 999  adam4adam.com 1 2 999  Lotus.com 1 2 999  cybermen.com 1 2 999  gaydar.fr  1 2 999  gay.com 1 2 999  Other 1 2 999 | |  |  | |
|  | Did you receive HIV counseling and testing at the Clinic de Halles in 2013? | Yes  No  Don’t know  Refuse to answer | | 1  2  888  999 |  | |
|  | Did you receive an STI consultation or treatment at the Clinic de Halles in 2013? | Yes  No  Don’t know  Refuse to answer | | 1  2  888  999 |  | |
|  | Did you receive HIV counseling and testing at the Clinic de Soutoura in 2013? | Yes  No  Don’t know  Refuse to answer | | 1  2  888  999 |  | |
|  | Did you receive an STI consultation or treatment at the Clinic de Soutoura in 2013? | Yes  No  Don’t know  Refuse to answer | | 1  2  888  999 |  | |
| 1. ***MALE SEX PARTNERS*** | | | | | | |
| **This next set of questions is about male sex partners and experiences you have had with them. While some people have had a lot of sexual partners, others have not, so questions may or may not apply to you. Please answer these questions as accurately as possible. To start, I will ask you about sexual experiences within the past 6 months.**  **For this next set of questions, we will refer to different types of sex partners. We will use the definitions below to define the types of sex partners. We will refer to these definitions of main partner, casual sex partners, and commercial partner in all remaining questions in the questionnaire that ask about type of sex partner. A handout will also be available during the interview that includes these definitions.**  **A main partner is the one you have sex with regularly and most often. A main sex partner is someone you are committed to, for example your spouse, live-in sex partner, or boyfriend. There is no payment or exchange of goods or services for sex with these partners.**  **A casual male partner is a man you have sex with but don’t feel committed to. Casual sex partners are people you have sex with occasionally or only once. There is no exchange of money or other things for sex with casual partners.**  **A commercial partner may have given you money or something for sex, or you may have given him money or something for sex.** | | | | | | |
|  | In the last 6 months, how many men did you have oral or anal sex with?  *Please give your best estimate.* | [__ [__|__]    Don’t know  Refuse to answer | | 888  999 | If answer is 0, recheck eligibility and stop interview if ineligible. You must enter 'Refuse to respond' for Questions E71-M236. Then complete Questions T03-T05 before dismissing the participant. | |
|  | In the last 6 months, how many men did you have anal sex with?  *Please give your best estimate.* | [__ [__|__]  Max: Q70  Don’t know  Refuse to answer | | 888  999 | Skip if Q54=2.  If 0, skip to Q76.. | |
|  | Of these *(response Q71)* men, how many were main partners? | [__ [__|__]  Max: Q71  Don’t know  Refuse to answer | | 888  999 | Skip if Q54=2. | |
|  | Of the *(response Q71)* men you had anal sex with in the last six months, how many were casual partners? | [__ [__|__]  Max: Q71  Don’t know  Refuse to answer | | 888  999 | Skip if Q54=2. | |
|  | Of the *(response Q71)* men you had anal sex with in the last six months, how many did you give or pay something for sex? | [__ [__|__]  Max: Q71  Don’t know  Refuse to answer | | 888  999 | Skip if Q54=2. | |
|  | Of the *(response Q71)* men you had anal sex with in the last six months, how many gave you money, goods or services for sex? | [__ [__|__]  Max: Q71  Don’t know  Refuse to answer | | 888  999 | Skip if Q54=2. | |
| **Check 1: Before continuing with the questionnaire, please make sure that the sum of the responses for Questions E72, E73, E74 and E75 is not greater than the response for Question E71.** | | | | | | |
| **Now I’m going to ask you a few questions about your experiences with your most recent male sex partners in the last 6 months.** | | | | | | |
|  | What kind of partner is the most recent man you’ve had oral or anal sex with? He could be a main, casual or commercial partner.  CHECK ONLY ONE | Main partner  Casual partner  Commercial – I paid him  Commercial – He paid me  Don’t know  Refuse to answer | | 1  2  3  4  888  999 |  | |
|  | Do you have a sexual relationship with this person, meaning you have had sex repeatedly with this person? | Yes  No  Don’t know  Refuse to answer | | 1  2  888  999 |  | |
|  | As far as you know, during the time you were having a sexual relationship with this partner did this person have sex with other people? | Definitely did not  Probably did not  Probably did  Definitely did  Don’t know  Refuse to answer | | 1  2  3  4  888  999 |  | |
|  | During the time you were having a sexual relationship with this person did you have sex with other people? | Yes  No  Don’t know  Refuse to answer | | 1  2  888  999 |  | |
|  | The last time you had sex with this person, did you or your partner drink alcohol before having sex? | I did  My partner did  We both did  Don’t know  Refuse to answer | | 1  2  3  888  999 |  | |
|  | The last time you had sex with this person, did you or your partner take drugs to get high before you had sex? | I did  My partner did  We both did  Don’t know  Refuse to answer | | 1  2  3  888  999 |  | |
|  | The last time you had sex with this person, did either of you wear a condom? | Yes  No  Don’t know  Refuse to answer | | 1  2  888  999 |  | |
|  | Think about the last time you did not use a condom. What was the main reason you did not use a condom?  CHECK ONLY ONE. | I didn’t have one  I don’t like them/I didn’t think of it  My partner objected  I didn’t feel at risk because I’m in a monogamous relationship  Other  Don’t know  Refuse to answer | | 1  2  3  4  777  888  999 |  | |
|  | With this sex partner, did you ever disclose each other’s HIV status? | Yes – only I disclosed  Yes – only my partner disclosed  We both disclosed  No  Don’t know  Refuse to answer | | 1  2  3  4  888  999 | If 1, 2,3, 888 and 999 skip to Q86. | |
|  | If no, why not?  DO NOT READ ANSWERS.  CHECK ONLY ONE | I thought he was negative  I thought he was positive  Not comfortable bringing it up  Did not want to ruin the mood  It was not important to me  Did not want to disclose my status  Other  Don’t know  Refuse to answer | | 1  2  3  4  5  6  777  888  999 |  | |
|  | What was his HIV status?  DO NOT READ ANSWERS.  CHECK ONLY ONE | He told me he was negative  He told me he was positive  I think he was negative  I think he was positive  Don’t know  Refuse to answer | | 1  2  3  4  888  999 |  | |
|  | In the last six months, did you ever have anal sex where you were the insertive (active) partner with him? | Yes  No  Don’t know  Refuse to answer | | 1  2  888  999 | Skip if Q54=2.  If 2, skip to Q91. | |
|  | In the last six months, did you ever have unprotected sex with him when you were the insertive (active) partner? | Yes  No  Don’t know  Refuse to answer | | 1  2  888  999 |  | |
|  | The last time you were the insertive (active) partner with him, did you use a condom the entire time? | Yes  No  Don’t know  Refuse to answer | | 1  2  888  999 |  | |
|  | Did you use a lubricant the last time you were the insertive (active) partner with him? | Yes  No  Don’t know  Refuse to answer | | 1  2  888  999 |  | |
|  | Have you ever been the receptive (passive) partner with him? | Yes  No  Don’t know  Refuse to answer | | 1  2  888  999 | If 2, skip to Q95. | |
|  | Did you ever have unprotected sex with him when you were the receptive (passive) partner? | Yes  No  Don’t know  Refuse to answer | | 1  2  888  999 |  | |
|  | The last time you were the receptive (passive) partner with him, did you use a condom the entire time? | Yes  No  Don’t know  Refuse to answer | | 1  2  888  999 |  | |
|  | Did you use a lubricant the last time you were the receptive (passive) partner with him? | Yes  No  Don’t know  Refuse to answer | | 1  2  888  999 |  | |
| 1. ***FEMALE SEX PARTNERS*** | | | | | | |
| **This next set of questions is about female sex partners and experiences you have had with them. To start, I will ask you about sexual experiences with females in the past 6 months.** | | | | | | |
|  | In the last 6 months, how many women did you have oral, vaginal or anal sex with? | [__|__]  None  Don’t know  Refuse to answer | | 0  888  999 | Only if Q51=1.  If 0, go to Q103. | |
|  | In the last 6 months, how many women did you have vaginal or anal sex with? | [__|__]  None  Don’t know  Refuse to answer  Max : Q95 | | 0  888  999 | Skip if Q95=0.  If 0, go to Q101. | |
|  | Of the *(response Q96)* women you had vaginal or anal sex with, how many were main partners? | [__|__]  None  Don’t know  Refuse to answer | | 0  888  999 | Skip if Q51=2. | |
|  | Of the *(response Q96)* women you had vaginal or anal sex with, how many were casual partners? | [__|__]  None  Don’t know  Refuse to answer | | 0  888  999 | Skip if Q51=2. | |
|  | Of the *(response Q96)* women you had vaginal or anal sex with, how many did you give money, goods, or services for sex? | [__|__]  None  Don’t know  Refuse to answer | | 0  888  999 | Skip if Q51=2. | |
|  | Of the *(response Q96)* women you had vaginal or anal sex with, how many gave you money, goods or services for sex? | [__|__]  None  Don’t know  Refuse to answer | | 0  888  999 | Skip if Q51=2.  Control: 96=97+98+99+100 | |
| **Check 2: Before continuing with the questionnaire, please make sure that the sum of the responses for Questions F97, F98, F99 and F100 is equal to the response for Question F96.** | | | | | | |
| **Now I’m going to ask you a few questions about your experiences with your most recent female sex partner.** | | | | | | |
|  | What kind of partner is the most recent woman you’ve had vaginal or anal sex with? She could be a main, casual or commercial partner.  CHECK ONLY ONE | Main partner  Casual partner  Commercial – I paid her  Commercial – She paid me  Don’t know  Refuse to answer | | 1  2  3  4  888  999 | Skip if Q51=2. | |
|  | The last time you had vaginal or anal sex with this partner, was a condom used the entire time? | Yes  No  Refuse to answer | | 1  2  999 | Skip if Q51=2. | |
| 1. ***CONDOMS AND LUBRICANTS***   **The next set of questions is about your use of condoms and lubricants in the last 6 months.** | | | | | | |
|  | Where do you usually get condoms?  DO NOT READ ANSWERS.  CHECK ALL THAT APPLY. | Shop or supermarket  Pharmacy or clinic  NGO/organization  Friends  Sex partner  Other  Refuse to answer | 1  2  3  4  5  777  999 | | |  |
|  | What is your favorite brand of condoms?  DO NOT READ ANSWERS.  CHECK ALL THAT APPLY. | Durex  Prudence  Manix  Hot Rubber  Protector/USAID  Inotek  Karma Sutra  Le Soft  Fresh Feeling  Belle Vie  Domino  Bravo  Other  Refuse to answer | 1  2  3  4  5  6  7  8  9  10  11  12  777  999 | | |  |
|  | Did you use free condoms in the last 6 months? | Yes  No  I have never received free condoms  Refuse to answer | 1  2  3  999 | | | If 2, 3, or 999, go to 107. |
|  | Do you think getting these free condoms made you more likely to use condoms during sex? | Yes  No  Don’t know  Refuse to answer | 1  2  888  999 | | |  |
|  | In the last 6 months have you ever been unable to get a condom when you need one? | Yes, I have been unable  No, I have always been able  Refuse to answer | 1  2  999 | | | If 2 or 999 go to Q110. |
|  | Why can’t you get a condom every time you need one?  DO NOT READ ANSWERS.  CHECK ALL THAT APPLY. | Costs too much  The place is too far away  The place is closed  Embarrassed to buy condom  Don’t know where to get condoms  Other  Don’t know  Refuse to answer | 1  2  3  4  5  777  888  999 | | |  |
|  | In the last 6 months, what are some reasons you couldn’t get condoms when you needed them?  DO NOT READ ANSWERS.  CHECK ALL THAT APPLY. | Can always get condoms  Costs too much  Not convenient  Clinic does not provide them  Embarrassed to get condoms  Do not know where to get condoms  Other  Refuse to answer | 1  2  3  4  5  6  777  999 | | |  |
|  | In the last 6 months, have you received information on condom use and safe sex? For example, through an outreach program or health clinic. | Yes  No  Refuse to answer | 1  2  999 | | |  |
|  | In the last 6 months, did you ever have a condom break during anal sex with a man? | Yes  No  Refuse to answer | 1  2  999 | | | Skip if Q71=0, 888 or 999. |
|  | Did you use lubricant the time that it broke? | Yes  No  Refuse to answer | 1  2  999 | | | Skip if Q71=0, 888 or 999. |
|  | Do you ever use more than one condom at a time? | Yes  No  Refuse to answer | 1  2  999 | | |  |
|  | Could you ask your main sex partner to use a condom if you wanted? | Yes  No  Refuse to answer | 1  2  999 | | |  |
|  | Under what circumstances do you tend not to use condoms during anal sex? With anal sex we mean a penis enters a person’s anus.  PROBE ALL. | y n dk nr  When I’m drunk or high 1 2 888 999  When I am afraid to ask my partner to use a condom or they refuse  1 2 888 999  When having sex with a regular partner  1 2 888 999  When having sex with a non-regular partner 1 2 888 999  When I am the insertive (top) partner  1 2 888 999  When I am the receptive (bottom) partner  1 2 888 999 When the person does not ejaculate inside me 1 2 888 999  Other 1 2 888 999 |  | | |  |
|  | Are you more likely to use a condom when a man inserts his penis into your anus (butt) or when you put your penis in his or equally likely? | When his penis is in me  When my penis is in him/her  Equally likely  Don’t know  Refuse to answer | 1  2  3  888  999 | | |  |
| **Some people use lubricants during anal sex. Lubricants make your penis or your partner’s penis more slippery and easier to insert into the anus. Lubricants also prevent the condom from breaking. Now I will ask you some questions about your use of lubricants in the last 6 months.** | | | | | | |
|  | In the past 6 months, have you used a lubricant during anal sex with a man? | Yes  No  Don’t know  Refuse to answer | 1  2  888  999 | | | Skip if Q71=0,888,999.  If 2, skip to 124. |
|  | In the last six months, which lubricants did you use during anal sex?  PROBE ALL | Y N NR  Saliva 1 2 999  Petroleum jelly (vaseline, pommade)  1 2 999  Water based lubricant (vendome, durex, etc ) 1 2 999  Beurre de karite 1 2 999  Lait corporel, hand lotion  1 2 999  Vaginal gel 1 2 999  Baby oil 1 2 999  Butter, blue band, cooking oil  1 2 999  Other 1 2 999 |  | | |  |
|  | In the last 6 months, where did you usually get water-based lubricants?  CHECK ALL THAT APPLY | Shop or supermarket  Pharmacy or clinic  NGO/organization  Friends  Sex partner  Other  Refuse to answer | 1  2  3  4  5  777  999 | | | Skip if respondent selects ‘No’ for Water based lubricant for Q118. |
|  | Are you able to get water-based lubricants when you need them? | Yes  No  Don’t know  Refuse to answer | 1  2  888  999 | | | Skip if respondent selects ‘No’ for Water based lubricant for Q118. |
|  | In the past 6 months, how frequently did you use water-based lubricant when having anal sex?  READ ANSWERS ALOUD.  CHECK ONLY ONE | Always Sometimes  Never  Refuse to answer | 1  2  3  999 | | | Skip if respondent selects ‘No’ for Water based lubricant for Q118.  Skip if Q71=0,888,999.  If 1, skip to Q123. |
|  | What is the main reason you do not always use a water-based lubricant during anal sex?  DO NOT READ ANSWERS.  CHECK ONLY ONE | Can’t get them easily/too expensive  Do not like lubricants  Partner doesn’t like them  I’ve never heard of it  I’m ashamed/embarrassed to buy it because it is associated with homosexuals  Other  Don’t know  Refuse to answer | 1  2  3  4  5  777  888  999 | | | Skip if Q121=1. |
|  | In the last 12 months, have you been given “packets” of lubricant for free? For example, through an outreach program or health clinic. | Yes  No  Don’t know  Refuse to answer | 1  2  888  999 | | |  |
| 1. ***SEX WORK***   **Receiving money, goods or services for sex** | | | | | | |
| **Now I am going to ask you some questions about if you have received money, goods or services to have sex.** | | | | | | |
|  | In the past 6 months, what did you receive in exchange for sex?  READ ANSWERS ALOUD. CHOOSE ALL THAT APPLY | Y N NR  Money 1 2 999  Goods 1 2 999  Services 1 2 999  Other 1 2 999 |  | | | Only if Q75≠0, 888 or 999 |
|  | How old were you when you first had sex with someone in exchange for money, goods or services? | Years: |__|__|  Max: Age of participant (Q7)  Don’t know  Refuse to answer | 888  999 | | | Only if Q75≠0, 888 or 999 |
|  | What is the main reason you started selling sex for money, goods or services? | Needed money, goods services to help the family  Needed money to pay a debt  was forced  Like to do it/pleasure/self-esteem  Friends/family were doing it  Other  Don’t know  Refuse to answer | 1  2  3  4  777  888  999 | | | Only if Q75≠0, 888 or 999 |
|  | In the past 6 months have you been given money, goods, or services to have sex from men, women or both? | Men  Women  Both  Refuse to answer | 1  2  3  999 | | | Only if Q75≠0, 888 or 999 |
|  | For how many years have you been selling sex for money, goods or services?  (*Probe if value suggests sex work started at age <13 years*) | Years: |__|__|  Less than a year  Don’t know  Refuse to answer | 0  888  999 | | | Only if Q75≠0, 888 or 999 |
|  | In which commune(s) of Bamako do you sell sex?  CHECK ALL THAT APPLY | Commune 1  Commune 2  Commune 3  Commune 4  Commune 5  Commune 6  Suburbs  Refuse to answer | 1  2  3  4  5  6  7  999 | | | Only if Q75≠0, 888 or 999 |
|  | In general, where do you usually find your male sex clients?  DO NOT READ RESPONSES  CHECK ALL THAT APPLY | School/university campus  Concert, Club, Bar, Restaurant  Private place  Religious organization  Spa/fitness center/beauty salon  Internet  River bank  Street  Other  Refuse to answer | 1  2  3  4  5  6  7  8  777  999 | | | Only if Q75≠0, 888 or 999 |
|  | Where do you usually *have* sex with your male clients?  DO NOT READ RESPONSES  CHECK ALL THAT APPLY | My or someone else’s home  Other private venue  Bar/ Hotel  Club  Open space (Park, field, river bank, etc.)  Work place  Other  Refuse to answer | 1  2  3  4  5  6  777  999 | | | Only if Q75≠0, 888 or 999 |
|  | Who usually decides where to have sex? | You  Client Both  Pimp  Other  Don’t know  Refuse to answer | 1  2  3  4  777  888  999 | | | Only if Q75≠0, 888 or 999 |
|  | Is selling sex your main source of income? | Yes  No  Don’t know  Refuse to answer | 1  2  888  999 | | | Only if Q75≠0, 888 or 999 |
|  | How many male clients did you have in the past week? | |__|__|  Don’t know  Refuse to answer | 888  999 | | | Only if Q75≠0, 888 or 999.  If 0, go to Q136. |
|  | With how many of those (response Q134) male clients did you use condoms? | |__|__|  Max: Q134  Don’t know  Refuse to answer | 888  999 | | | Only if Q75≠0, 888 or 999. |
|  | In the last 6 months, has a client abused or threatened you? | Yes  No  Don’t know  Refuse to answer | 1  2  888  999 | | | Only if Q75≠0, 888 or 999 |
|  | In the last 6 months, has a client forced you to have sex? | Yes  No  Don’t know  Refuse to answer | 1  2  888  999 | | | Only if Q75≠0, 888 or 999 |
| 1. ***STIGMA, VIOLENCE AND MENTAL HEALTH***   **Now we will ask you some questions about stigma, sexual violence and mental health. Remember that everything you say is confidential.** | | | | | | |
|  | Do you think it is illegal to have sex with other men in Mali? | Yes, it is illegal  No, it is not illegal  Don’t know  Refuse to answer | 1  2  888  999 | | |  |
|  | Have you ever been arrested because you have sex with men? | Yes  No  Don’t know  Refuse to answer | 1  2  888  999 | | |  |
|  | Have your friends or family left you because you have sex with men? | Yes  No  Don’t know  Refuse to answer | 1  2  888  999 | | |  |
|  | Have you been terminated from a job because you have sex with men? | Yes  No  Don’t know  Refuse to answer | 1  2  888  999 | | |  |
|  | Have you ever been blackmailed by someone because you have sex with other men? | Yes  No  Don’t know  Refuse to answer | 1  2  888  999 | | |  |
|  | Have you ever been treated unfairly or denied health care because you have sex with men? | Yes  No  Don’t know  Refuse to answer | 1  2  888  999 | | |  |
|  | When you seek health care, do you feel you need to hide that you have sex with men? | Yes  No  Don’t know  Refuse to answer | 1  2  888  999 | | |  |
|  | Have you ever suffered any harassment or abuse because you have sex with other men? | Yes  No  Refuse to answer | 1  2  999 | | | If 2, skip to Q148. |
|  | What sort of harassment or abuse was that?  PROBE ALL | Y n nr  Physical (beaten) 1 2 999  Verbal (threats, insults) 1 2 999  Moral (isolation, exclusion) 1 2 999  Sexual (forced to have sexual contact)  1 2 999  Other 1 2 999 |  | | |  |
|  | Who harassed or abused you this way?  PROBE ALL | Y n nr  Family members  1 2 999  Sex partner 1 2 999  Friends, other people I know 1 2 999  ‘Authority figure’ (religious leader, employer, teacher)  1 2 999  Health care worker 1 2 999  Strangers 1 2 999  Prison inmate 1 2 999  Uniformed service personnel 1 2 999  Other 1 2 999 |  | | |  |
|  | Have you ever been forced to have sex against your will? | Yes  No  Refuse to answer | 1  2  999 | | | If 2, skip to Q150. |
|  | Who forced you to have sex against your will?  DO NOT READ ANSWERS.  CHECK ALL THAT APPLY. | Family members  Sex partner  Friends, other people I know  ‘authority figure’ (religious leader, employer, teacher)  Health care worker  Strangers  Prison inmate  Uniformed service personnel  Other  Refuse to answer | 1  2  3  4  5  6  7  8  777  999 | | |  |
| **Next we will ask you some questions on how you feel about your sexual attraction to men.** | | | | | | |
|  | I have tried to stop being attracted to men in general. | Strongly disagree  Disagree  Neutral  Agree  Strongly agree  Don’t know  Refuse to answer | 1  2  3  4  5  888  999 | | |  |
|  | If someone offered me the chance to be completely heterosexual, I would accept the chance. | Strongly disagree  Disagree  Neutral  Agree  Strongly agree  Don’t know  Refuse to answer | 1  2  3  4  5  888  999 | | |  |
|  | I wish I weren't gay/bisexual. | Strongly disagree  Disagree  Neutral  Agree  Strongly agree  Don’t know  Refuse to answer | 1  2  3  4  5  888  999 | | |  |
|  | I feel that being gay/bisexual is a personal shortcoming for me. | Strongly disagree  Disagree  Neutral  Agree  Strongly agree  Don’t know  Refuse to answer | 1  2  3  4  5  888  999 | | |  |
|  | I would like to get professional help in order to change my sexual orientation from gay/bisexual to heterosexual. | Strongly disagree  Disagree  Neutral  Agree  Strongly agree  Don’t know  Refuse to answer | 1  2  3  4  5  888  999 | | |  |
| **SOCIAL COHESION**  **The next several questions are about your social life and your relationships with other men who have sex with men. Please mark if you strongly disagree, disagree, are neutral, agree, or strongly agree with the statements.** | | | | | | |
|  | You can count on other MSM if you need to borrow money. | Strongly disagree  Disagree  Neutral  Agree  Strongly agree  Don’t know  Refuse to answer | 1  2  3  4  5  888  999 | | |  |
|  | You can count on other MSM to accompany you to the doctor or hospital. | Strongly disagree  Disagree  Neutral  Agree  Strongly agree  Don’t know  Refuse to answer | 1  2  3  4  5  888  999 | | |  |
|  | You can count on other MSM if you need to talk about your problems. | Strongly disagree  Disagree  Neutral  Agree  Strongly agree  Don’t know  Refuse to answer | 1  2  3  4  5  888  999 | | |  |
|  | You can count on other MSM if you need somewhere to stay. | Strongly disagree  Disagree  Neutral  Agree  Strongly agree  Don’t know  Refuse to answer | 1  2  3  4  5  888  999 | | |  |
|  | In the past 6 months, have you negotiated with or stood up against a non-MSM in order to help a fellow MSM? | Yes  No  Don’t know  Refuse to answer | 1  2  888  999 | | |  |
| 1. ***KNOWLEDGE, OPINIONS, AND ATTITUDES TOWARDS HIV/AIDS***   **Now we are going to ask you some questions about your general knowledge, opinions and attitudes about HIV.** | | | | | | |
|  | Is it possible for a healthy-looking person to have HIV? | Yes  No  Don’t know  Refuse to answer | 1  2  888  999 | | |  |
|  | Can you reduce the risk of HIV by having just one uninfected sex partner who has no other partners? | Yes  No  Don’t know  Refuse to answer | 1  2  888  999 | | |  |
|  | Can a person reduce the risk of getting HIV by using a condom every time they have sex? | Yes  No  Don’t know  Refuse to answer | 1  2  888  999 | | |  |
|  | Can a person get HIV from mosquito bites? | Yes  No  Don’t know  Refuse to answer | 1  2  888  999 | | |  |
|  | Can a person get HIV by sharing food with someone who is infected? | Yes  No  Don’t know  Refuse to answer | 1  2  888  999 | | |  |
|  | Do you think it is more likely for someone to get HIV through sex with men or women? | Men  Women  About the same  Don’t know  Refuse to answer | 1  2  3  888  999 | | |  |
|  | If a condom is not used, what kind of sex puts you at the most risk for HIV?  CHECK ONLY ONE | Manual sex  Oral sex  Vaginal sex  Anal sex  Don’t know  Refuse to answer | 1  2  3  4  888  999 | | |  |
|  | If a condom is not used, what is the second riskiest kind of sex?  CHECK ONLY ONE | Manual sex  Oral sex  Vaginal sex  Anal sex  Don’t know  Refuse to answer | 1  2  3  4  888  999 | | |  |
|  | Compared to vaginal sex, how important is it to use condoms for *anal* sex?  CHECK ONLY ONE | Less important  Equally important  More important  Don’t know  Refuse to answer | 1  2  3  888  999 | | |  |
|  | If a condom is not used, what kind of anal sex puts you at most risk for HIV?  CHECK ONLY ONE | Active (top) anal sex  Passive (bottom) anal sex  Both have same risk  Both have no risk  Don’t know  Refuse to answer | 1  2  3  4  888  999 | | |  |
|  | Do you know anyone with HIV/AIDS? | Yes  No  Don’t know  Refuse to answer | 1  2  888  999 | | | If 2, skip to Q172. |
|  | Do you know any HIV-positive men who have sex with men? | Yes  No  Don’t know  Refuse to answer | 1  2  888  999 | | |  |
|  | Do you know any men who have sex with men who have died from HIV/AIDS? | Yes  No  Don’t know  Refuse to answer | 1  2  888  999 | | |  |
|  | Do you think it is possible you have HIV? | Yes  No  I already know I have HIV  Don’t know  Refuse to answer | 1  2  3  888  999 | | |  |
|  | How likely do you think it is that you will get infected with HIV in the next year? | Extremely unlikely  Somewhat unlikely  Somewhat likely  Extremely likely  Don’t know  Refuse to answer | 1  2  3  4  888  999 | | |  |
|  | Is there an effective treatment for HIV/AIDS? | Yes  No  Don’t know  Refuse to answer | 1  2  888  999 | | |  |
|  | Do you agree with this statement: “*I am not as careful about HIV and sex now because there is better treatment for AIDS*” | Agree  Disagree  Don’t know  Refuse to answer | 1  2  888  999 | | |  |
| 1. ***HIV INFORMATION AND SERVICES***   **Now we are going to ask some questions about the way and kind of information you have received about HIV and the information you want to receive.** | | | | | | |
|  | Who or what has the most influence over your sexual risk behaviors?  DO NOT READ ANSWERS.  CHECK ONLY ONE | Friends  Family  Sexual partner  Social norms  Religion  HIV awareness materials  Health care agents/providers  Other  Don’t know  Refuse to answer | 1  2  3  4  5  6  7  777  888  999 | | |  |
|  | Do you think the HIV messages you have seen apply to men who have sex with men? | Yes  No  Don’t know  Refuse to answer | 1  2  888  999 | | |  |
|  | Do you think the HIV messages you have seen apply to you? | Yes  No  Don’t know  Refuse to answer | 1  2  888  999 | | | If 1,888 or 999 go to Q181. |
|  | Why do you think the messages do not apply to you? | They are not about MSM  They are not about anal sex  There is a woman in the message/picture  They are about pregnant women  Other  Don’t know  Refuse to answer | 1  2  3  4  777  888  999 | | |  |
|  | Where do you like to get information about HIV from?  DO NOT READ ANSWERS.  CHECK ALL THAT APPLY. | Radio  Television  Newspaper  Internet  Telephone/SMS  Brochure  Friends  Family  Sex partners  Health care providers  Peer educator/outreach worker  Religious leader  Don’t know  Refuse to answer | 1  2  3  4  5  6  7  8  9  10  11  12  888  999 | | |  |
|  | What HIV-related topics do you want to learn more about?  DO NOT READ ANSWERS.  CHECK ALL THAT APPLY. | How HIV is transmitted  How to prevent HIV  How to treat HIV  How to use a condom  Talking to partner about condom use  Abstinence  Monogamy  Safe injections  Others  Don’t know  Refuse to answer | 1  2  3  4  5  6  7  8  777  888  999 | | |  |
| **We will now ask you about services that peer educators or outreach workers may have given you. A peer educator is someone like you who has been trained in HIV prevention. An outreach worker is someone employed by an organization, government or private agency, who might provide these same services to people like you.** | | | | | | |
|  | Has a peer educator or outreach worker ever talked to you about HIV? | Yes  No  Don’t know  Refuse to answer | 1  2  888  999 | | | If 2, skip to Q186. |
|  | How long ago did a peer educator or outreach worker talk to you about HIV? | In the last 30 days  In the last 3 months  In the last year  Longer than a year ago  Don’t know  Refuse to answer | 1  2  3  4  888  999 | | |  |
|  | What did you receive the last time you met a peer educator or outreach worker?  CHECK ALL THAT APPLY. | Nothing  Condoms  Lubricants  Pamphlet or brochure  Medicines  Other  Refuse to answer | 1  2  3  4  5  777  999 | | |  |
| 1. ***HIV TESTING, CARE AND TREATMENT***   **Now I am going to ask you some questions about your HIV testing, care and treatment experience. Remember that your answers are confidential.** | | | | | | |
|  | Have you ever had an HIV test? | Yes  No  Refuse to answer | 1  2  999 | | | If 1, skip to Q188.  If 999, skip to Q216. |
|  | What is the main reason you have never tested for HIV? | I feel I am not at risk for HIV  Fear of positive result  No money to get tested  No time to get tested  Stigma by health care workers  Other  Refuse to answer | 1  2  3  4  5  777  999 | | | Go to Q216. |
|  | When did you last test for HIV? | In the last 6 months  Between 7 and 12 months ago  More than 12 months ago  Don’t know  Refuse to answer | 1  2  3  888  999 | | |  |
|  | Why did you decide to have your most recent HIV test?  DO NOT READ ANSWERS.  CHECK ALL THAT APPLY. | Because I wanted to know  Felt sick/suspected  New partner  Know someone newly infected  Health provider/worker advised  Employer pressure  Premarital testing  Other  Refuse to answer | 1  2  3  4  5  6  7  777  999 | | |  |
|  | Which of the following best describes the counseling you received? | Respectful, caring, understanding  Disrespectful, uncaring, stigmatizing, uncomfortable  Neither respectful nor disrespectful  Don’t know  Refuse to answer | 1  2  3  888  999 | | |  |
|  | Did you receive your HIV test result? | Yes  No  Refuse to answer | 1  2  999 | | | If 2 or 999, skip to Q216. |
|  | Who did you tell about your most recent HIV test result?  DO NOT READ ANSWERS.  CHECK ALL THAT APPLY. | No one  Sex partner  Family member  Friend  Healthcare provider  Other  Refuse to answer | 1  2  3  4  5  777  999 | | |  |
|  | What was the result of your most recent HIV test? | Negative  Positive  Unclear / neither positive nor negative  Refuse to answer | 1  2  3  999 | | | If 1,3 or 999, skip to Q216 |
|  | Have you ever been harassed or stigmatized by other men who have sex with men because you have HIV? | Yes  No  Refuse to answer | 1  2  999 | | | Only if 193=2 |
| **Many positive people register with a health care provider to get care. By “care” we mean someone goes for check-ups to a health care provider or gets ARVs for their HIV infection. The next few questions are about the first time you saw a provider for your HIV.** | | | | | | |
|  | After you tested HIV positive, did you ever visit a health care provider to discuss your HIV? | Yes  No  Don’t know  Refuse to answer | 1  2  888  999 | | | If 1,888, 999, skip to 197a |
|  | What is the main reason you have never visited a health care provider for HIV care?  SELECT ONLY ONE | Feel healthy  Stigma, don’t want others to know  Cost or transportation problems  Poor attitude of health care workers  Waiting time or clinic hours not good  Other  Don’t know  Refuse to answer | 1  2  3  4  5  777  888  999 | | | All, skip to Q216. |
| **197a..** | Were you being monitored for HIV care at the Clinique de Halles in 2013? | Yes  No  Don’t know  Refuse to answer | 1  2  888  999 | | | Only if Q195 = 1, 888 or 999 |
|  | Do you still regularly access health care or check-ups for your HIV? | Yes, still in care  No, I stopped receiving care/going to the clinic  Don’t know  Refuse to answer | 1  2  888  999 | | | If 1, 888, 999 go to 199 |
|  | What is the main reason you no longer go to a clinic for HIV care?  SELECT ONLY ONE | Feel healthy  Stigma, don’t want others to know  Cost or transportation problems  Poor attitude of health care workers  Waiting time or clinic hours not good  Other  Don’t know  Refuse to answer | 1  2  3  4  5  777  888  999 | | |  |
| **Cotrimoxazole or cotrim is a medicine recommended for people with HIV, even if they have not started treatment. It helps prevent certain infections but it does not treat HIV. We will ask you a few questions about cotrimoxazole.** | | | | | | |
|  | Have you ever taken cotrimoxazole? | Yes  No  Don’t know  Refuse to answer | 1  2  888  999 | | | If 1 skip to Q201.  If 888, 999 skip to 202 |
|  | What is the main reason you don’t take cotrimoxazole? | Feel healthy  Fear of stigma  Not offered by clinic  Clinic too far  Cost  No longer effective  Other  Don’t know  Refuse to answer | 1  2  3  4  5  6  777  888  999 | | | All skip to 202 |
|  | Do you still take cotrimoxazole? | Yes  No  Don’t know  Refuse to answer | 1  2  888  999 | | |  |
| **We will now ask you a few questions about your “CD4” or “T-cell” count. The CD4 count tells how sick you are with HIV or how weak your immune system is and if you need to take antiretrovirals.** | | | | | | |
|  | Have you ever had your CD4 tested? | Yes  No  Don’t know  Refuse to answer | 1  2  888  999 | | | If 2, 888, 999 go to 206 |
|  | When did your care provider last test your CD4 count? | In the last 6 months  Between 7 and 12 months ago  More than 12 months ago  Don’t know  Refuse to answer | 1  2  3  888  999 | | |  |
|  | Did you receive your CD4 test result at the same visit or later? | Same visit  Next day  Later visit  Don’t know  Refuse to answer | 1  2  3  888  999 | | |  |
|  | What was the result of your last CD4 count? | More than 500  Between 350 and 499  Between 200 and 349  Below 200  Don’t know  Refuse to answer | 1  2  3  4  888  999 | | |  |
| **We will now ask you some questions on anti-retroviral treatment, also called ARVs, to treat HIV.** | | | | | | |
|  | Have you ever taken ARVs to treat your HIV? | Yes  No  Don’t know  Refuse to answer | 1  2  888  999 | | |  |
|  | What is the main reason you never started taking ARVs?  SELECT ONLY ONE | My CD4 count is still high  My CD4 count is unknown  I am on a waiting list to start  Health care provider told me its too early to start  Other reason  Don’t know  Refuse to answer | 1  2  3  4  777  888  999 | | | Only if Q206=2 |
|  | You said other. Which of the following is the main reason you never started taking ARVs? | No clinic near me offers it  Health care provider refused to give me ARVs  Fear of stigma or that others find out  Costs too much  Other  Don’t know  Refuse to answer | 1  2  3  4  777  888  999 | | | Only if Q207=777 |
|  | Do you currently take ARVs? | Yes  No  Don’t know  Refuse to answer | 1  2  888  999 | | | Only if Q206=1, 888 or 999 |
|  | While taking ARVs, do you or did you use any of the following services?  CHECK ALL THAT APPLY. | Mobile phone text reminders  Treatment support group  Food or money support  Outreach worker or peer educator  None of these  Refuse to answer | 1  2  3  4  5  999 | | | Only if Q209=1, 888, or 999 |
|  | What is the main reason why you stopped taking ARVs? | Felt healthy  Too many side effects  God will heal me  I didn’t want to take ARVs anymore  It was difficult for me to get ARVs  Other  Refuse to answer | 1  2  3  4  5  777  999 | | | Only if Q209=2 |
| **The next few questions are about tuberculosis or TB. All people with HIV should be “screened” for TB. With “screening,” we mean health care staff ask you about if you have a cough, fever, or sudden weight loss.** | | | | | | |
|  | Have you ever been screened for TB? | Yes  No  Don’t know  Refuse to answer | 1  2  888  999 | | | If 2, 888 or 999 skip to 216 |
|  | When did they screen you for TB? | Before I tested HIV-positive  After I tested HIV-positive  Both before and after I tested HIV-positive  Don’t know  Refuse to answer | 1  2  3  888  999 | | |  |
|  | What was the result of your last TB test? | Tb-negative  Tb-positive  Indeterminate  Don’t know  Refuse to answer | 1  2  3  888  999 | | |  |
|  | Did you get treatment for TB? | Yes  No  Don’t know  Refuse to answer | 1  2  888  999 | | |  |
| 1. ***SEXUALLY TRANSMITTED INFECTIONS (IST)***   **Now I want to ask you some questions about your sexual health. Remember that your answers are confidential.** | | | | | | |
|  | If you had a sexual health problem tomorrow, where would you go for health care?  DO NOT READ RESPONSES  SELECT ONLY ONE | Pharmacy  Hospital  Auto-prescription  Traditional medicine  Marabout  Soutoura  ARCAD/SIDA  Clinic Halles  Private clinic  Public clinic  Other  Don’t know  Refuse to answer | 1  2  3  4  5  6  7  8  9  10  777  888  999 | | |  |
|  | In the last 12 months have you visited a doctor or nurse? | Yes  No  Don’t know  Refuse to answer | 1  2  888  999 | | | If 2, 888, 999 go to Q219 |
|  | Did they check to see if you have any sexually transmitted infections? | Yes  No  Don’t know  Refuse to answer | 1  2  888  999 | | |  |
|  | Have you had any abnormal **discharge from your penis** in the past 12 months?  SHOW PICTURE | Yes  No  Don’t know  Refuse to answer | 1  2  888  999 | | |  |
|  | Have you had an **ulcer or sore on or near your penis** in the past 12 months?  SHOW PICTURE | Yes  No  Don’t know  Refuse to answer | 1  2  888  999 | | |  |
|  | In the last 12 months, have you found an ulcer or sore on or near your partner’s penis or vagina? | Yes  No  Don’t know  Refuse to answer | 1  2  888  999 | | |  |
|  | Have you had an **anal ulcer** or sore in the past 12 months?  SHOW PICTURE | Yes  No  Don’t know  Refuse to answer | 1  2  888  999 | | |  |
|  | Have you had **anal discharge** in the past 12 months? | Yes  No  Don’t know  Refuse to answer | 1  2  888  999 | | |  |
|  | Have you had **anal warts** in the past 12 months?  SHOW PICTURE | Yes  No  Don’t know  Refuse to answer | 1  2  888  999 | | | If all Q219-Q224 = 2, skip to question T03. |
|  | If you were having sex while you had these symptoms, did you inform the person(s) with whom you were having sex?  *With sex we mean either vaginal sex or anal sex. With vaginal sex we mean a penis enters a vagina. With anal sex we mean a penis enters a person’s anus.* | Yes  No  Don’t know  Refuse to answer | 1  2  888  999 | | |  |
|  | While you had these symptoms, did you abstain from having sex? | Yes  No  Don’t know  Refuse to answer | 1  2  888  999 | | |  |
|  | While you had these symptoms, did you always use a condom during sex? | Yes  No  Don’t know  Refuse to answer | 1  2  888  999 | | |  |
|  | Why did you not inform your sexual partner(s) that you might have an STI?  DO NOT READ ANSWERS.  CHECK ONLY ONE. | Afraid partner would be upset  Afraid partner would become violent  Afraid partner would leave me  Afraid partner would tell others  Did not know how to locate partner  Did not feel it was necessary to discuss  I received treatment  Other  Refuse to answer | 1  2  3  4  5  6  7  777  999 | | | Skip if Q225=1. |
|  | Did the person(s) you were having sex with get treated for this problem? | Yes  No  Don’t know  Refuse to answer | 1  2  888  999 | | |  |
|  | Did you see a healthcare provider because of these problems? | Yes  No  Don’t know  Refuse to answer | 1  2  888  999 | | |  |
|  | Did you go to a pharmacy to get treatment? | Yes  No  Don’t know  Refuse to answer | 1  2  888  999 | | |  |
|  | How long did it take you to go see the healthcare provider since the appearance of these problems?  DO NOT READ ANSWERS.  CHECK ONLY ONE. | Less than one week  More than one week, less than one month  More than one month  Don’t know  Refuse to answer | 1  2 3  888  999 | | | Only if Q230=1 |
|  | Why did you not get treatment for this problem?  DO NOT READ ANSWERS.  CHECK ONLY ONE. | Thought it would go away itself  Do not know where to get it  Concerned about confidentiality  Negative attitude of health care workers  Cost  Distance  Other  Refuse to answer | 1  2  3  4  5  6  777  999 | | | Skip if Q230=1 & Q231=1 |
|  | Did the healthcare provider tell you that you had a sexually transmitted infection? | Yes  No  Don’t know  Refuse to answer | 1  2  888  999 | | | Only if Q230=1 |
|  | The last time you had a sexually transmitted infection did you feel comfortable getting treatment from health care staff? | Yes  No  Don’t know  Refuse to answer | 1  2  888  999 | | | Only if Q230=1 |
|  | The last time you were treated for a sexually transmitted disease did you feel stigmatized by the health care staff? | Yes  No  Don’t know  Refuse to answer | 1  2  888  999 | | | Only if Q230=1 |

| ***Questionnaire parameters*** | | | |
| --- | --- | --- | --- |
| **T03** | Study ID  *Please respond to this question before dismissing the study participant.*  *Each study participant is assigned a four-digit sequential study ID number: XZZZ, where ""X"" designates the study site (A or B), and ""ZZZ"" is the running number starting with participant 001.* |  |  |
| **T04** | Was the interview completed without incident? | Yes  No | 1  2 |
| **T05** | Interview non-completed-reason  (If T04=2) | Fear of being seen  Disturbing questions  No time  No motivation  Ineligible  Other  Refuse to answer | 1  2  3  4  5  777  999 |
