# Supplementary material for: An urgent need for HIV testing among men who have sex with men and transgender women in Bamako, Mali: Low awareness of HIV infection and viral suppression among those living with HIV
Source: PLoS One. 2018 Nov 12;13(11):e0207363. doi: 10.1371/journal.pone.0207363 (PMC6231666; doi:10.1371/journal.pone.0207363)
Supplement: S2 Appendix — (PDF) [file pone.0207363.s002.pdf]

QUESTIONNAIRE

ETUDE TERIYA

Avant de commencer l'entrevue, s'il vous plaît assurez-vous que le formulaire de sélection a été complété et enregistré sur la liste de contrôle et que le consentement éclairé a été obtenu et enregistré sur la liste de contrôle.

Introduction: « *Bonjour, je m'appelle .... Je vais vous orienter dans cet entretien. Vous pouvez refuser de répondre à certaines questions et vous pouvez interrompre l'entretien à tout moment. Cependant le fait de répondre à toutes les questions garantirait la réussite de cette enquête. Si vous ne connaissez pas la réponse exacte à une question, essayez au mieux de donner votre avis. Pour la plupart des questions, choisissez la réponse qui vous convient le mieux. Nous vous indiquerons à chaque fois lorsqu'une question donne droit à plus d'une réponse. Si vous avez besoin d'aide, il faudra seulement l'exprimer. Toutes vos réponses seront confidentielles. Nous ne vous demanderons pas votre nom ou toute autre information permettant de vous identifier. »*

Paramètres du Questionnaire			
**T01**	Code de l'enquêteur (initiaux)<br><br>*S'il vous plaît entrer seulement vos initiales (deux lettres).*	☐☐	
**T02a**	Site de l'étude	Site A<br>Site B	1<br>2
**T02b**	Identifiant de l'étude<br><br>*A chaque participant à l'étude, il est attribué un numéro d'identification séquentiel à l'étude de quatre chiffres: XZZZ, où «X» désigne le site de l'étude (A ou B) et «ZZZ» est le numéro d'ordre commençant par le participant 001.*<br><br>*L'identifiant de l'étude ne doit contenir la lettre indiquant le site d'étude et les trois chiffres attribués au participant.*	☐☐☐☐	
**start_time _date**	Heure et date du début de l'entretien *(automatique)*		
**end_time_ date**	Heure et date de la fin de l'entretien *(automatique)*		

No.	QUESTIONS ET ELEMENTS DE FILTRAGE DES QUESTIONS	CATEGORIES DE CODAGE		SAUTER LES QUESTIONS
	A. INFORMATION DU RECRUITER **D'abord, je vais te poser des questions sur la personne qui t'a remis le coupon et sur d'autres hommes qui ont des rapports sexuelles avec d'autres hommes que tu connais à Bamako. Rappelles-toi que toutes tes réponses sont confidentielles**			
**1.**	Qu'est ce qui décrit le mieux la manière dont tu connais la personne qui t'a remis ce coupon?	Membre de la famille<br>Partenaire Sexuelle<br>Ami<br>Connaissance<br>Etranger<br>Autre<br>Refuse de répondre	1<br>2<br>3<br>4<br>5<br>777<br>999	Si la réponse est 1, 3, 4, 5, 777, 999 allez à Q3.
**2.**	Quel type de partenaire sexuel est cette personne ?<br><br>*L'enquêteur peut préciser les types de partenaires sexuels en utilisant le document disponible au cours de l'entrevue.*	Partenaire principal<br>Partenaire occasionnel<br>Professionnel du Sexe – Je l'ai payé<br>Client – Il m'a payé<br>Ne sait pas<br>Refuse de répondre	1<br>2<br>3<br>4<br>888<br>999	
**3.**	Connais-tu cette personne depuis quelques jours, quelques mois ou des années ?	Jours<br>Mois<br>Années<br>Ne sait pas<br>Refuse de répondre	1<br>2<br>3<br>888<br>999	
**4.**	Combien de fois avez-vous vu cette personne les 30 derniers jours?	Nombre: |__|__]<br>Entre: 0-30<br>Ne sait pas<br>Refuse de répondre	 <br>888<br>999	
**5.**	Ton coupon a-t-il été échangé contre quelque chose ?	Non, rien<br>Argent ou biens<br>Faveurs sexuelles<br>Autre<br>Refuse de répondre	1<br>2<br>3<br>777<br>999	
**6.**	Pourquoi avez-vous décidé de participer à cette étude?<br><br>NE PAS LIRE LES REPONSES A HAUTE VOIX<br>CHOISIR UNE SEULE REPONSE	Je m'intéresse au sujet<br>Je veux connaître mon statut sérologique<br>Je veux de l'argent pour participer<br>J'ai subi des pressions pour participer<br>Je veux aider la communauté<br>Autre<br>Refuse de répondre	1<br>2<br>3<br>4<br>5<br>777<br>999	
	B. CARACTERISTIQUES DU CONTEXTE **Maintenant, je vais poser quelques questions d'ordre général sur vous-même. Rappelez-vous que toutes vos réponses sont confidentielles.**			
**7.**	Quel âge avez-vous ?	Age (années)<br>Refuse de répondre	[_I_]<br>999	

No.	QUESTIONS ET ELEMENTS DE FILTRAGE DES QUESTIONS	CATEGORIES DE CODAGE		SAUTER LES QUESTIONS
**8.**	De quelle nationalité êtes-vous?<br><br>NE PAS LIRE LES REPONSES A HAUTE VOIX - CHOISIR UNE SEULE REPONSE	Malienne<br>Ivoirienne<br>Burkinabé<br>Sénégalais<br>Ghanéenne<br>Guinéenne<br>Libérienne<br>Togolaise<br>Béninoise<br>Nigériane<br>Autres pays africains<br>Autres pays non africains<br>Refuse de répondre	1<br>2<br>3<br>4<br>5<br>6<br>7<br>8<br>9<br>10<br>11<br>12<br>999	
**9.**	Lequel de ces pays ou quels autres endroits au Mali avez-vous visité ces 6 derniers mois?<br><br>NE PAS LIRE LES REPONSES A HAUTE VOIX<br>CHOISIR TOUTES LES REPONSES QUI S'APPLIQUENT	Une autre ville au Mali (en dehors de Bamako)<br>Cote d'Ivoire<br>Burkina Faso<br>Ghana<br>Guinée<br>Libéria<br>Togo<br>Bénin<br>Nigéria<br>Autres pays africains<br>Autres pays non africains<br>Refuse de répondre	1<br>2<br>3<br>4<br>5<br>6<br>7<br>8<br>9<br>10<br>11<br>999	
**10.**	Dans quelle commune de Bamako vivez-vous ?<br><br>NE PAS LIRE LES REPONSES A HAUTE VOIX<br>CHOISIR UNE SEULE REPONSE	Commune 1<br>Commune 2<br>Commune 3<br>Commune 4<br>Commune 5<br>Commune 6<br>Périphéries<br>Refuse de répondre	1<br>2<br>3<br>4<br>5<br>6<br>7<br>999	Si la réponse est 1, 2, 3, 4, 5, 6 ou 999 allez à Q12.
**11.**	Dans quel quartier en périphérie de Bamako vivez-vous?<br><br>NE PAS LIRE LES REPONSES A HAUTE VOIX<br>CHOISIR UNE SEULE REPONSE	Kati<br>Kalabankoro<br>Senou<br>Moribabougou<br>Sangarebougou<br>Titibougou<br>Baguineda<br>Safo<br>Sirakoro Megatana<br>Niamana<br>Samaya<br>Dialakorodji<br>Dialakoroba<br>Sanakoroba<br>Autre<br>Refuse de répondre	1<br>2<br>3<br>4<br>5<br>6<br>7<br>8<br>9<br>10<br>11<br>12<br>13<br>14<br>777<br>999	

No.	QUESTIONS ET ELEMENTS DE FILTRAGE DES QUESTIONS	CATEGORIES DE CODAGE		SAUTER LES QUESTIONS
**12.**	Pouvez-vous lire et écrire?	Ne peut pas lire et écrire<br>Peut lire seulement<br>Peut lire et écrire<br>Ne sait pas<br>Refuse de répondre	1<br>2<br>3<br>888<br>999	
**13.**	Quel est votre niveau d'études le plus élevé?	Jamais allé à l'école<br>Alphabétisé<br>Fondamentale<br>Secondaire<br>Enseignement supérieur<br>Post-Université<br>Refuse de répondre	1<br>2<br>3<br>4<br>5<br>6<br>999	Si la réponse est 1 allez à Q15.
**14.**	Quelle est la dernière année ou la dernière classe que vous avez fréquentée?	Année/Classe<br>Ne sait pas<br>Refuse de répondre	[_I_]<br>888<br>999	
**15.**	Avez-vous fréquenté une école technique ou professionnelle?	Oui<br>Non<br>Refuse de répondre	1<br>2<br>999	
**16.**	Quelle est votre principale occupation ou profession actuelle?<br><br>NE PAS LIRE LES REPONSES A HAUTE VOIX<br>CHOISIR UNE SEULE REPONSE	Sans emploi<br>Etudiant<br>Ouvrier journalier, vendeur de rue, marchand ambulant<br>Mécanicien, ouvrier d'usine, ouvrier<br>Professionnel/enseignant/banquier/comptable<br>Coiffeur<br>Serveur/gérant de bar/hôtel, etc.<br>Personne en uniforme<br>Professionnel du sexe<br>Artisan/Musicien/danseur/interprète de chants<br>Chauffeur<br>Cultivateur<br>Sécurité (société de gardiennage)<br>Fonctionnaire de l'Etat<br>Autre<br>Refuse de répondre	1<br>2<br>3<br>4<br>5<br>6<br>7<br>8<br>9<br>10<br>11<br>12<br>13<br>14<br>777<br>999	
**17.**	Combien (argent) avez-vous gagné le mois précédent?<br><br>CHOISIR UNE SEULE REPONSE	< 25 000 CFA<br>25 000 – 49 999 CFA<br>50 000 – 149 999 CFA<br>150 000 – 299 999 CFA<br>≥ 300 000 CFA<br>Ne sait pas<br>Refuse de répondre	1<br>2<br>3<br>4<br>5<br>888<br>999	
**18.**	De quelle religion êtes-vous?<br><br>NE PAS LIRE LES REPONSES A HAUTE VOIX<br>CHOISIR UNE SEULE REPONSE	Islam<br>Christianisme<br>Animisme<br>Une autre religion<br>Sans religion<br>Refuse de répondre	1<br>2<br>3<br>4<br>5<br>999	

No.	QUESTIONS ET ELEMENTS DE FILTRAGE DES QUESTIONS	CATEGORIES DE CODAGE		SAUTER LES QUESTIONS
**19.**	Quelle est votre situation matrimoniale actuelle?<br><br>CHOISIR UNE SEULE REPONSE	Jamais marié<br>Marié (à une seule femme)<br>Marié (à plus d'une femme)<br>Divorcé, Séparé, ou veuf<br>Refuse de répondre	1<br>2<br>3<br>4<br>999	
**20.**	Dans quel type de maison vivez-vous?<br><br><br>NE PAS LIRE LES REPONSES A HAUTE VOIX<br>CHOISIR UNE SEULE REPONSE	Maison<br>Appartement<br>Dortoir<br>Centre communautaire<br>Rue/sans domicile<br>Autre<br>Refuse de répondre	1<br>2<br>3<br>4<br>5<br>777<br>999	
**21.**	Avez-vous un endroit régulier pour dormir la nuit?	Oui<br>Non<br>Refuse de répondre	1<br>2<br>999	
**22.**	Au cours des 2 dernières semaines, combien de fois avez-vous eu peu d'intérêt ou de plaisir à faire les choses?	Pas du tout<br>Plusieurs jours<br>Plus de la moitié des jours<br>Presque tous les jours<br>Refuse de répondre	1<br>2<br>3<br>4<br>999	
**23.**	Au cours des 2 dernières semaines, combien de fois avez-vous eu le sentiment d'être triste, déprimé ou désespéré?	Pas du tout<br>Plusieurs jours<br>Plus de la moitié des jours<br>Presque tous les jours<br>Refuse de répondre	1<br>2<br>3<br>4<br>999	
**24.**	Combien de fois buvez-vous une boisson contenant de l'alcool?	Jamais<br>Une fois par mois ou moins<br>2-3 fois par mois<br>2-3 fois par semaine<br>Quatre fois ou plus par semaine<br>Refuse de répondre	1<br>2<br>3<br>4<br>5<br>999	Si la réponse est 1 allez à Q27
**25.**	Combien de verres standards buvez-vous au cours d'une journée ordinaire?<br><br>En verre standard, nous entendons une bouteille de bière (350 ml), un verre de vin (150 ml) ou 45 ml de liqueur.	1 à 2<br>3 à 4<br>5 à 6<br>7 à 9<br>≥10<br>Refuse de répondre	1<br>2<br>3<br>4<br>5<br>999	
**26.**	Combien de fois buvez-vous six verres ou plus en une seule occasion?	Jamais<br>Moins une fois par mois<br>Une fois par mois<br>Une fois par semaine<br>Chaque jour ou presque<br>Refuse de répondre	1<br>2<br>3<br>4<br>5<br>999	

No.	QUESTIONS ET ELEMENTS DE FILTRAGE DES QUESTIONS	CATEGORIES DE CODAGE		SAUTER LES QUESTIONS
**27.**	Certaines personnes prennent des drogues ou des médicaments non injectables pour le plaisir ou pour des raisons autres que celles recommandées par un médecin. Avez-vous pris des médicaments non injectables pour le plaisir ou autres raisons ces 6 derniers mois?	Oui<br>Non<br>Refuse de répondre	1<br>2<br>999	Si la réponse est 2 ou 999 allez à Q29.
**28.**	Au cours des 6 derniers mois, avez-vous utilisé:<br><br>FAITES UN SONDAGE SUR CHAQUE ELEMENT DE LA REPONSE	Oui Non NSP NR<br>De la colle (A inhaler) 1 2 888 999<br>Tabac 1 2 888 999<br>Du pétrole (Inhaler) 1 2 888 999<br>De la Marijuana (Haschisch) 1 2 888 999<br>De l'Héroïne (Nono) 1 2 888 999<br>De la Cocaïne 1 2 888 999<br>Des Amphétamines 1 2 888 999<br>Des médicaments 1 2 888 999<br>Autre 1 2 888 999		
**29.**	Certaines personnes s'injectent des drogues avec une seringue pour le plaisir ou pour des raisons autres que celles recommandées par un médecin. Dans votre vie, avez-vous déjà injecté des médicaments autres que ceux prévus pour vous? En injection, je veux dire si vous avez utilisé des drogues illégales ou illicites avec une aiguille, soit par injection dans la veine, l'injection sous la peau), ou par injection dans le muscle.	Oui<br>Non<br>Refuse de répondre	1<br>2<br>999	Si la réponse est 2 ou 999 allez à Q31.
**30.**	Avez-vous déjà partagé une aiguille ou une seringue avec quelqu'un d'autre ces 6 derniers mois?	Oui<br>Non<br>Refuse de répondre	1<br>2<br>999	
	C. <u>TAILLE DU RESEAU</u><br>**Maintenant, je vais te poser des questions sur le nombre de personnes que tu connais.**			
**31.**	Combien d'hommes connaissez-vous qui ont des rapports sexuels avec d'autres hommes?<br><br>OBTENEZ LA BONNE ESTIMATION DU PARTICIPANT	XXX<br><br>Nombre [__|__|__]<br><br>Refuse de répondre	 <br><br><br>999	Si le participant répond avec '0,' sondez avec la question suivante: "Que dire de la personne qui vous a donné un coupon?"<br><br>Si 0 ou 999, allez à Q37

No.	QUESTIONS ET ELEMENTS DE FILTRAGE DES QUESTIONS	CATEGORIES DE CODAGE		SAUTER LES QUESTIONS
**32.**	Combien de ces XXX hommes que vous connaissez habitent ou travaillent à Bamako ou dans ses quartiers en périphérie?<br><br>OBTENEZ LA BONNE ESTIMATION DU PARTICIPANT	YYY<br><br>Nombre [__|__|__]<br>Max : XXX<br><br>Refuse de répondre	 <br><br><br><br>999	Si 0 ou 999, allez à Q37
**33.**	Combien de ces YYY hommes ont 18 ans ou plus ?<br><br>OBTENEZ LA BONNE ESTIMATION DU PARTICIPANT	ZZZ<br><br>Nombre [__|__|__]<br>Max : YYY<br><br>Refuse de répondre	 <br><br><br><br>999	Si 0 ou 999, allez à Q37
**34.**	Combien de ces ZZZ hommes avez-vous vu ces 30 derniers jours ?<br><br>OBTENEZ LA BONNE ESTIMATION DU PARTICIPANT	TTT<br><br>Nombre [__|__|__]<br>Max : ZZZ<br><br>Refuse de répondre	 <br><br><br><br>999	Si 0 ou 999, allez à Q37.
**35.**	De ces TTT hommes, combien voulez-vous envisager d'inviter à participer à cette enquête?<br><br>OBTENEZ LA BONNE ESTIMATION DU PARTICIPANT	Nombre [__|__|__]<br>Min : 000<br>Max : TTT<br><br>Refuse de répondre	 <br><br><br>999	Si 0 ou 999, allez à Q37
**36.**	Chez combien de ces TTT hommes que vous avez vu ces 30 derniers jours avez-vous le numéro dans votre téléphone portable?<br><br>OBTENEZ LA BONNE ESTIMATION DU PARTICIPANT	Nombre [__|__|__]<br>Max : TTT<br><br>Refuse de répondre	 <br><br>999	Si 0 ou 999, allez à Q37
**37.**	Sans compter la personne qui vous a remis ce coupon, combien MSM que vous connaissez ont déjà participé à cette étude?	Nombre [__|__|__]<br>Refuse de répondre	 999	
	**D. *IDENTITE SEXUELLE ET EXPERIENCES SEXUELLES***<br><br>**Les prochaines questions portent sur votre identité et orientation sexuelle. Toutes vos réponses sont confidentielles.**<br><br>**Par homosexuel/gay, nous voulons dire quelqu'un qui s'identifie comme attiré par quelqu'un du même sexe. Par bisexuel nous voulons dire quelqu'un qui s'identifie comme étant attiré à la fois par les hommes et les femmes. Par hétérosexuel, nous voulons dire un individu qui s'identifie comme attiré par le sexe opposé.**			
**38.**	Vous identifiez-vous souvent comme homme, femme, travesti ou quelque chose d'autre ?	Homme<br>Femme<br>Travesti<br>Quelque chose d'autre/autre<br>Refuse de répondre	1<br>2<br>3<br>4<br>999	

No.	QUESTIONS ET ELEMENTS DE FILTRAGE DES QUESTIONS	CATEGORIES DE CODAGE		SAUTER LES QUESTIONS
**39.**	Vous vous décririez comme étant :<br><br>LIRE LES REPONSES- CHOISIR UNE SEULE REPONSE	Gay/Homosexuel<br>Bisexuel<br>Hétérosexuel<br>Autre<br>Ne sait pas<br>Refuse de répondre	1<br>2<br>3<br>777<br>888<br>999	
**40.**	Quel sexe vous attire le plus sur le plan sexuel?<br><br>NE PAS LIRE LES REPONSES A HAUTE VOIX - CHOISIR UNE SEULE REPONSE	Seulement ou majoritairement masculin<br>Les deux sexes à part égale<br>Seulement ou majoritairement féminin<br>Ne sait pas<br>Refuse de répondre	1<br>2<br>3<br>888<br>999	
**41.**	En plus de vos partenaires masculins, à qui avez-vous dit que vous êtes attiré par des hommes?<br><br>CHOISIR TOUTES LES REPONSES QUI CONVIENNENT	Aucun<br>Membres de famille<br>Epouse ou partenaire<br>Amis qui ne sont pas gay, lesbienne ou bisexuel<br>Amis qui sont gay, lesbienne ou bisexuel<br>Prestataire de soins de santé<br>Autre<br>Ne sait pas<br>Refuse de répondre	1<br>2<br>3<br>4<br><br>5<br>6<br>777<br>888<br>999	
**42.**	A propos de votre famille: Diriez-vous que votre famille accepte que vous êtes attiré par des hommes, ne le sait pas, ou vous rejette pour cela?	M'accepte<br>Ne le sait pas<br>Me rejette<br>Refuse de répondre	1<br>2<br>3<br>999	Si Q38≠3 (pas trans), allez à Q51
**43.**	Quel est votre sexe de naissance?	Homme<br>Femme<br>Hermaphrodite/Ambiguë<br>Refuse de répondre	1<br>2<br>3<br>999	SEULEMENT POUR CEUX QUI S'IDENTIFIENT COMME TRANS (Q38=3)
**44.**	Durant les 6 derniers mois, avez-vous déjà vécu comme une femme? En vivant comme une femme, je veux dire en se présentant et en s'habillant comme une femme.	Oui<br>Non<br>Refuse de répondre	1<br>2<br>999	SEULEMENT POUR CEUX QUI S'IDENTIFIENT COMME TRANS (Q38=3)
**45.**	Parmi les suivants, à qui avez-vous parlé de votre identité transgénique?<br><br>CHOISIR TOUTES LES REPONSES QUI CONVIENNENT	Amis travestis<br>Amis Gay/Lesbienne qui ne sont pas travestis<br>Amis hétérosexuels qui ne sont pas travestis<br>Famille<br>Epouse<br>Prestataire de soins de santé<br>Autre<br>Ne sait pas<br>Refuse de répondre	1<br>2<br><br>3<br>4<br>5<br>6<br>777<br>888<br>999	SEULEMENT POUR CEUX QUI S'IDENTIFIENT COMME TRANS (Q38=3)
**46.**	Avez-vous déjà utilisé des hormones pour changer votre corps?	Oui<br>Non<br>Refuse de répondre	1<br>2<br>999	SEULEMENT POUR CEUX QUI S'IDENTIFIENT COMME TRANS (Q38=3)<br><br>Si 2 or 999, allez à Q48.

No.	QUESTIONS ET ELEMENTS DE FILTRAGE DES QUESTIONS	CATEGORIES DE CODAGE		SAUTER LES QUESTIONS
**47.**	Combien de fois avez-vous eu des injections d'hormone les 6 derniers mois?	Jamais<br>Au moins une fois par jour<br>Une fois par semaine<br>Une fois par mois<br>Refuse de répondre	1<br>2<br>3<br>4<br>999	SEULEMENT POUR CEUX QUI S'IDENTIFIENT COMME TRANS (Q38=3)
**48.**	En plus des hormones, avez-vous eu d'autres procédures de renforcement ou de transition physique?	Oui<br>Non<br>Refuse de répondre	1<br>2<br>999	SEULEMENT POUR CEUX QUI S'IDENTIFIENT COMME TRANS (Q38=3)
**49.**	Pensez à la dernière fois que vous avez eu des rapports sexuels avec un partenaire masculin. Est-ce que ce partenaire savait que vous étiez travesti ?	Oui<br>Non<br>Ne sait pas<br>Refuse de répondre	1<br>2<br>888<br>999	SEULEMENT POUR CEUX QUI S'IDENTIFIENT COMME TRANS (Q38=3)
**50.**	Est-ce que vous lui avez dit que vous êtes travesti?	Oui<br>Non<br>Refuse de répondre	1<br>2<br>999	SEULEMENT POUR CEUX QUI S'IDENTIFIENT COMME TRANS (Q38=3)
**Expériences sexuelles**<br><br>**Les prochaines questions portent sur l'histoire de votre vie sexuelle. Cela inclut le rapport sexe vaginal et anal. Avec le sexe vaginal, nous entendons un pénis qui pénètre dans le vagin. Avec le sexe anal, nous entendons un pénis qui pénètre dans l'anus d'une personne (butt).**				
**51.**	Avez-vous déjà eu des rapports sexuels oraux, vaginaux ou anaux avec une femme?	Oui<br>Non<br>Refuse de répondre	1<br>2<br>999	Si 2 ou 999 allez à Q53.
**52.**	Quel âge aviez-vous lors de votre premier rapport oral, vaginal ou anal avec une femme?	Age (années)<br>Max : Q7<br>Ne sait pas<br>Refuse de répondre	[_I_]<br>888<br>999	
**53.**	Avez-vous déjà eu des rapports oraux avec un homme?	Oui<br>Non<br>Refuse de répondre	1<br>2<br>999	

No.	QUESTIONS ET ELEMENTS DE FILTRAGE DES QUESTIONS	CATEGORIES DE CODAGE		SAUTER LES QUESTIONS
**54.**	Avez-vous déjà eu des rapports anaux avec un homme?	Oui<br>Non<br>Refuse de répondre	1<br>2<br>999	Si Q53 & Q54 = 2, vérifiez l'éligibilité et arrêtez l'entrevue si la personne n'est pas éligible. Vous devez entrer «Refuse de répondre» pour les questions D55-M236. Puis complétez Questions T03-T05 avant de laisser sortir le participant.
**55.**	S'il vous plaît penser à la première fois que vous aviez des rapports sexuels avec un homme.<br>Quel âge aviez-vous lors de votre premier rapport avec un homme?	Age (années)<br>Max : Q7<br>Ne sait pas<br>Refuse de répondre	[_I_]<br>888<br>999	
**56.**	Approximativement quel âge avait votre partenaire?<br><br>*S'il vous plait donnez votre meilleure estimation.*	10-14 ans<br>15-19 ans<br>20-24 ans<br>25-29 ans<br>30-34 ans<br>35-39 ans<br>40 ans ou plus<br>Ne sait pas<br>Refuse de répondre	1<br>2<br>3<br>4<br>5<br>6<br>7<br>888<br>999	
**57.**	Est-ce qu'il a vous payé ou donné quelque chose en échange des relations sexuelles?	Oui<br>Non<br>Ne sait pas<br>Refuse de répondre	1<br>2<br>888<br>999	
**58.**	Avez-vous payé ou lui donner quelque chose en échange des relations sexuelles?	Oui<br>Non<br>Ne sait pas<br>Refuse de répondre	1<br>2<br>888<br>999	

No.	QUESTIONS ET ELEMENTS DE FILTRAGE DES QUESTIONS	CATEGORIES DE CODAGE		SAUTER LES QUESTIONS
**59.**	Comment décririez-vous la manière dont vous l'aviez connu?<br><br>NE PAS LIRE LES REPONSES A HAUTE VOIX - CHOISIR UNE SEULE REPONSE	Ami / partenaire<br>Ami / connaissance / collègue<br>Parent<br>Etranger<br>'Autorité (responsable du gouvernement, leader religieux, professeur, employeur, militaires, policiers, gardiens de prison)<br>Autre<br>Ne sait pas<br>Refuse de répondre	1<br>2<br>3<br>4<br>5<br><br>777<br>888<br>999	
**60.**	Quel genre d'«autorité» était-il?	Professeur / enseignant<br>Leader religieux<br>Employeur<br>Militaire / policier<br>Gardien de prison<br>Autre<br>Ne sait pas<br>Refuse de répondre	1<br>2<br>3<br>4<br>5<br>777<br>888<br>999	Seulement si Q59=5
**61.**	Avez-vous déjà habité ensemble avec un partenaire de sexe masculin?	Oui<br>Non<br>Refuse de répondre	1<br>2<br>999	
**62.**	A part vos partenaires de sexe masculin, avez-vous parlé à quiconque de vos rapports sexuels avec des hommes?	Oui<br>Non<br>Ne sait pas<br>Refuse de répondre	1<br>2<br>888<br>999	Si 2, allez à Q64.
**63.**	A qui avez-vous parlé de vos rapports sexuels avec des hommes?<br><br>FAITES UN SONDAGE SUR CHAQUE ELEMENT DE LA REPONSE	Oui Non NR<br>Des amis MSM/Lesbien/Bi/Trans 1 2 999<br>Autres amis 1 2 999<br>Conjointe 1 2 999<br>Autres membres de la famille 1 2 999<br>Agents de santé 1 2 999<br>Autre 1 2 999		
**64.**	Avez-vous déjà offert quelque chose à un homme pour avoir des rapports sexuels avec lui? Par quelque chose, je veux dire de l'argent, des biens ou des faveurs.	Oui<br>Non<br>Ne sait pas<br>Refuse de répondre	1<br>2<br>888<br>999	
**65.**	Sur lequel de ces sites web avez-vous un compte ou un profil?<br><br>FAITES UN SONDAGE SUR CHAQUE ELEMENT DE LA REPONSE	Oui Non NR<br>gayromeo.com 1 2 999<br>draguenet.net 1 2 999<br>adam4adam.com 1 2 999<br>Lotus.com 1 2 999<br>cybermen.com 1 2 999<br>gaydar.fr 1 2 999<br>gay.com 1 2 999<br>Autre 1 2 999		

No.	QUESTIONS ET ELEMENTS DE FILTRAGE DES QUESTIONS	CATEGORIES DE CODAGE		SAUTER LES QUESTIONS
**66.**	Avez-vous bénéficié d'un conseille et dépistage VIH au niveau de la Clinique de Halles en 2013?	Oui<br>Non<br>Ne sait pas<br>Refuse de repondre	1<br>2<br>888<br>999	
**67.**	Avez-vous bénéficié d'une consultation ou un traitement IST au niveau de la Clinique de Halles en 2013?	Oui<br>Non<br>Ne sait pas<br>Refuse de repondre	1<br>2<br>888<br>999	
**68.**	Avez-vous bénéficié d'un conseille et dépistage VIH au niveau de la Clinique de Soutoura en 2013?	Oui<br>Non<br>Ne sait pas<br>Refuse de repondre	1<br>2<br>888<br>999	
**69.**	Avez-vous bénéficié d'une consultation ou un traitement IST au niveau de la Clinique de Soutoura en 2013?	Oui<br>Non<br>Ne sait pas<br>Refuse de repondre	1<br>2<br>888<br>999	

E. *PARTENAIRES DU SEXE MASCULIN*

La prochaine série de questions portera sur vos partenaires de sexe masculin et vos expériences avec eux. Alors que certaines personnes ont eu beaucoup de partenaires sexuels, d'autres en n'ont pas eu, donc certaines questions peuvent s'appliquer à vous ou pas. S'il vous plaît répondez à ces questions le plus correctement possible. Pour commencer, je vais vous poser des questions sur vos expériences sexuelles avec les hommes au cours des 6 derniers mois.

Pour cette prochaine série de questions, nous allons référer à différents types de partenaires sexuels. Nous allons utiliser les définitions suivants pour définir les types de partenaires sexuels. Nous ferons référence à ces définitions de partenaire principal, les partenaires sexuels occasionnels, et partenaire commercial dans toutes les autres questions dans le questionnaire qui posent des questions sur le type de partenaire sexuel. Un document sera également disponible pendant l'entrevue qui comprend ces définitions.

Un partenaire sexuel principal est celui avec qui vous avez régulièrement et le plus souvent des rapports sexuels. Un partenaire sexuel principal est quelqu'un avec qui vous êtes engagé, par exemple pourrait être votre conjoint, vie en partenariat sexuel, ou petit ami. Il n'y a pas de paiement ou d'échange de biens ou de services pour des rapports sexuels avec ces partenaires.

Un partenaire sexuel occasionnel est quelqu'un avec qui vous avez des relations sexuelles, mais avec qui vous ne vous sentez pas engagé. Les partenaires sexuels occasionnels sont des personnes avec qui vous avez des rapports sexuels occasionnellement ou seulement une fois. Il n'y a aucun échange d'argent ou d'autre chose contre des rapports sexuels avec des partenaires occasionnels.

Un partenaire commercial peut être un client (il vous a donné de l'argent ou des cadeaux en échange des rapports sexuels) ou un professionnel du sexe (vous lui avez donné de l'argent ou des cadeaux en échange des rapports sexuels).

No.	QUESTIONS ET ELEMENTS DE FILTRAGE DES QUESTIONS	CATEGORIES DE CODAGE		SAUTER LES QUESTIONS
**70.**	Au cours des 6 derniers mois, avec combien d'hommes avez-vous eu des rapports oraux ou anaux ?<br><br>*Veuillez donner votre meilleure estimation*	[__ [__|__]<br>Ne sait pas<br>Refuse de répondre	 888<br>999	Si la réponse est 0, vérifiez l'éligibilité et arrêter l'entrevue si la personne n'est pas éligible. Vous devez entrer «Refuse de répondre» pour les questions E71-M236. Puis complétez Questions T03-T05 avant de laisser sortir le participant.
**71.**	Au cours des 6 derniers mois, avec combien d'hommes avez-vous eu des rapports anaux?<br><br>*Veuillez donner votre meilleure estimation*	[__ [__|__]<br>Max : Q70<br>Ne sait pas<br>Refuse de répondre	 <br>888<br>999	Sautez si Q54=2.<br>Si 0, allez à Q95.
**72.**	Parmi ces *(réponse Q71)* hommes, combien ont été vos partenaires principaux?	[__ [__|__]<br>Max : Q71<br>Ne sait pas<br>Refuse de répondre	 <br>888<br>999	Sautez si Q54=2.
**73.**	Parmi les *(réponse Q71)* hommes avec qui vous avez eu des rapports anaux au cours des 6 derniers mois, combien ont été occasionnels?	[__ [__|__]<br>Max : Q71<br>Ne sait pas<br>Refuse de répondre	 <br>888<br>999	Sautez si Q54=2.
**74.**	Parmi les *(réponse Q71)* hommes avec qui vous avez eu des rapports anaux au cours des 6 derniers mois, combien avez-vous payé ou donné quelque chose en échange des rapports sexuels?	[__ [__|__]<br>Max : Q71<br>Ne sait pas<br>Refuse de répondre	 <br>888<br>999	Sautez si Q54=2.
**75.**	Parmi les *(réponse Q71)* hommes avec qui vous avez eu des rapports anaux au cours des 6 derniers mois, combien vous ont donné de l'argent, des biens ou des faveurs en échange des rapports sexuels?	[__ [__|__]<br>Max : Q71<br>Ne sait pas<br>Refuse de répondre	 <br>888<br>999	Sautez si Q54=2.

No.	QUESTIONS ET ELEMENTS DE FILTRAGE DES QUESTIONS	CATEGORIES DE CODAGE		SAUTER LES QUESTIONS
**Check 1 : Avant de continuer avec le questionnaire, s'il vous plaît assurez-vous que la somme des réponses pour les questions E72, E73, E74 et E75 n'est pas supérieure à la réponse pour la question E71.**				
**Maintenant, je vais vous poser quelques questions sur vos expériences sexuelles avec votre plus récent partenaire de sexe masculin au cours des 6 derniers mois.**				
**76.**	Quel type de partenaire est l'homme le plus récent avec qui vous avez eu des rapports oraux ou anaux? Il pourrait être un partenaire principal, occasionnel ou commercial.<br><br>COCHEZ UNE SEULE REPONSE	Partenaire principal<br>Partenaire occasionnel<br>Professionnel du Sexe – Je l'ai payé<br>Client – Il m'a payé<br>Ne sait pas<br>Refuse de répondre	1<br>2<br>3<br>4<br>888<br>999	
**77.**	Avez-vous une relation sexuelle avec cette personne, ce qui signifie que vous avez eu des relations sexuelles à plusieurs reprises avec cette personne?	Oui<br>Non<br>Ne sait pas<br>Refuse de répondre	1<br>2<br>888<br>999	
**78.**	Autant que vous le savez, pendant le temps que vous aviez une relation sexuelle avec ce partenaire n'avait il pas des relations sexuelles avec d'autres personnes?	N'a certainement pas<br>N'a probablement pas<br>Probablement fait<br>Certainement fait<br>Ne sait pas<br>Refuse de répondre	1<br>2<br>3<br>4<br>888<br>999	
**79.**	Pendant le temps que vous aviez une relation sexuelle avec cette personne, avez-vous eu des rapports sexuels avec d'autres personnes?	Oui<br>Non<br>Ne sait pas<br>Refuse de répondre	1<br>2<br>888<br>999	
**80.**	La dernière fois que vous avez eu des rapports sexuels avec cette personne, est-ce que vous ou votre partenaire avez bu de l'alcool avant d'avoir des rapports sexuels?	J'ai fait<br>Mon partenaire a fait<br>Nous avons tous deux fait<br>Ne sait pas<br>Refuse de répondre	1<br>2<br>3<br>888<br>999	
**81.**	La dernière fois que vous avez eu des rapports sexuels avec cette personne, avez-vous ou votre partenaire pris des médicaments pour se droguer avant de le faire?	J'ai fait<br>Mon partenaire a fait<br>Nous avons tous deux fait<br>Ne sait pas<br>Refuse de répondre	1<br>2<br>3<br>888<br>999	
**82.**	La dernière fois que vous avez eu des rapports sexuels avec cette personne, avez-vous ou votre partenaire porté un préservatif?	Oui<br>Non<br>Ne sait pas<br>Refuse de répondre	1<br>2<br>888<br>999	

No.	QUESTIONS ET ELEMENTS DE FILTRAGE DES QUESTIONS	CATEGORIES DE CODAGE		SAUTER LES QUESTIONS
**83.**	Pensez à la dernière fois que vous n'avez pas utilisé un préservatif. Quelle était la raison principale pour laquelle vous n'aviez pas utilisé un condom?<br><br>COCHEZ UNE SEULE REPONSE	Je n'ai pas eu un<br>Je ne les aime pas / je ne le pensais pas<br>Mon partenaire s'était opposé<br>Je ne me sentais pas en danger parce que je suis dans une relation monogame<br>Autre<br>Ne sait pas<br>Refuse de répondre	1<br>2<br>3<br>4<br>777<br>888<br>999	
**84.**	Avec ce partenaire sexuel, avez-vous ou votre partenaire divulgué à l'un ou l'autre son statut VIH?	Oui - que j'ai divulgué<br>Oui – que mon partenaire a divulgué<br>Nous avons tous deux divulgué<br>Non<br>Ne sait pas<br>Refuse de répondre	1<br>2<br>3<br>4<br>888<br>999	Si 1, 2,3,888 ou 999 sautez à Q86.
**85.**	Si non, pourquoi pas?<br><br>NE PAS LIRE LES REPONSES.<br>COCHEZ UNE SEULE REPONSE	Je pensais qu'il était séronégatif<br>Je pensais qu'il était séropositif<br>Je ne suis pas à l'aise quand j'aborde ce sujet<br>Je ne voulais pas gâter l'atmosphère<br>Ce n'était pas important pour moi<br>Je ne voulais pas révéler mon statut<br>Autre<br>Ne sait pas<br>Refuse de répondre	1<br>2<br>3<br>4<br>5<br>6<br>777<br>888<br>999	
**86.**	Quelle était son statut VIH?<br><br>NE PAS LIRE LES REPONSES.<br>COCHEZ UNE SEULE REPONSE	Il m'a dit qu'il était séronégatif<br>Il m'a dit qu'il était séropositif<br>Je pense qu'il est séronégatif<br>Je pense qu'il est séropositif<br>Ne sait pas<br>Refuse de répondre	1<br>2<br>3<br>4<br>888<br>999	
**87.**	Au cours des 6 derniers mois, avez-vous déjà eu des rapports anaux lors desquels vous étiez le partenaire « insertif » (actif) avec lui?	Oui<br>Non<br>Ne sait pas<br>Refuse de répondre	1<br>2<br>888<br>999	Sautez si Q54=2.<br>Si 2, allez à Q91.
**88.**	Au cours des 6 derniers mois, avez-vous déjà eu des rapports sexuels non protégés lors desquels vous étiez le partenaire « insertif » (actif) avec lui?	Oui<br>Non<br>Ne sait pas<br>Refuse de répondre	1<br>2<br>888<br>999	
**89.**	La dernière fois que vous étiez le partenaire « insertif » (actif), avez-vous utilisé un préservatif durant tout le temps de votre rapport sexuel?	Oui<br>Non<br>Ne sait pas<br>Refuse de répondre	1<br>2<br>888<br>999	
**90.**	Avez-vous utilisé un lubrifiant cette dernière fois que vous étiez le partenaire "insertif" (actif)?	Oui<br>Non<br>Ne sait pas<br>Refuse de répondre	1<br>2<br>888<br>999	

No.	QUESTIONS ET ELEMENTS DE FILTRAGE DES QUESTIONS	CATEGORIES DE CODAGE		SAUTER LES QUESTIONS
**91.**	Avez-vous déjà été « réceptif » (passif) avec ce partenaire?	Oui<br>Non<br>Ne sait pas<br>Refuse de répondre	1<br>2<br>888<br>999	Si 2, sautez à Q95.
**92.**	Avez-vous déjà eu des rapports sexuels non protégés au cours desquels vous étiez le partenaire réceptif (passif) avec lui?	Oui<br>Non<br>Ne sait pas<br>Refuse de répondre	1<br>2<br>888<br>999	
**93.**	La dernière fois que vous étiez le partenaire réceptif (passif) avec lui, avez-vous utilisé un préservatif durant tout le temps de votre rapport sexuel?	Oui<br>Non<br>Ne sait pas<br>Refuse de répondre	1<br>2<br>888<br>999	
**94.**	Avez-vous utilisé un lubrifiant cette dernière fois que vous étiez le partenaire « receptif » (passif) avec lui?	Oui<br>Non<br>Ne sait pas<br>Refuse de répondre	1<br>2<br>888<br>999	
	**F. *PARTENAIRES DU SEXE FEMININ***			
	**La prochaine série de questions portera sur vos partenaires de sexe féminins et vos expériences avec eux. Pour commencer, je vais vous poser des questions sur vos expériences sexuelles avec les femmes au cours des <u>6 derniers mois</u>.**			
**95.**	Au cours des 6 derniers mois, avec combien de femmes avez-vous eu des rapports oraux, vaginaux ou anaux ?	[__|__]<br>Aucune<br>Ne sait pas<br>Refuse de répondre	 0<br>888<br>999	Seulement si Q51=1.<br>Si 0, allez à IQ103.
**96.**	Au cours des 6 derniers mois, avec combien de femmes avez-vous eu des rapports vaginaux ou anaux?	[__|__]<br>Aucune<br>Ne sait pas<br>Refuse de répondre<br><br>Max : Q95	 0<br>888<br>999	Sautez si Q95=0.<br>Si 0, allez à IQ101.
**97.**	Parmi ces *(réponse Q96)* femmes avec qui vous avez eu des rapports vaginaux ou anaux, combien ont été vos partenaires principales?	[__|__]<br>Aucune<br>Ne sait pas<br>Refuse de répondre	 0<br>888<br>999	Sautez si Q51=2.
**98.**	Parmi ces *(réponse Q96)* femmes avec qui vous avez eu des rapports vaginaux ou anaux, combien ont été vos partenaires occasionnelles?	[__|__]<br>Aucune<br>Ne sait pas<br>Refuse de répondre	 0<br>888<br>999	Sautez si Q51=2.
**99.**	Parmi les *(réponse Q96)* femmes avec lesquelles vous avez eu des rapports vaginaux ou anaux, combien d'entre elles avez-vous donné de l'argent, des biens ou des services en échange des rapports sexuels?	[__|__]<br>Aucune<br>Ne sait pas<br>Refuse de répondre	 0<br>888<br>999	Sautez si Q51=2.

No.	QUESTIONS ET ELEMENTS DE FILTRAGE DES QUESTIONS	CATEGORIES DE CODAGE		SAUTER LES QUESTIONS
**100.**	Parmi les *(réponse Q96)* femmes avec lesquelles vous avez eu des rapports vaginaux ou anaux, combien d'entre elles vous ont donné de l'argent, des biens ou des services en échange des rapports sexuels?	[__|__]<br>Aucune<br>Ne sait pas<br>Refuse de répondre	 0<br>888<br>999	Sautez si Q51=2.<br><br>Contrôlez : 96=97+98+99+ 100
**Check 2 : Avant de continuer avec le questionnaire, s'il vous plaît assurez-vous que la somme des réponses pour les questions F97, F98, F99 et F100 est égale à la réponse pour la question F96.**				
**Maintenant, je vais vous poser quelques questions sur vos expériences avec votre plus récente partenaire de sexe féminin.**				
**101.**	Quel type de partenaire est la femme la plus récente avec qui vous avez eu des rapports vaginaux ou anaux? Elle pourrait être une partenaire principale, occasionnelle ou commerciale.<br><br>COCHEZ UNE SEULE REPONSE	Partenaire principale<br>Partenaire occasionnelle<br>Professionnelle du Sexe – Je l'ai payée<br>Client –Elle m'a payé<br>Ne sait pas<br>Refuse de répondre	1<br>2<br>3<br>4<br>888<br>999	Sautez si Q51=2.
**102.**	La dernière fois que vous avez eu un rapport sexuel avec cette partenaire, avez-vous utilisé un préservatif durant tout le temps de votre rapport ?	Oui<br>Non<br>Refuse de répondre	1<br>2<br>999	Sautez si Q51=2.
G. PRESERVATIFS ET LUBRIFIANTS<br>**La prochaine série de questions porte sur votre utilisation de préservatifs et de lubrifiants dans les 6 derniers mois.**				
**103.**	Où trouvez-vous habituellement des préservatifs?<br><br>NE PAS LIRE LES REPONSES A HAUTE VOIX - CHOISIR TOUTES LES REPONSES QUI S'APPLIQUENT	Boutique ou supermarché<br>Pharmacie ou une clinique<br>ONG / organisation<br>Amis<br>Partenaire sexuel<br>Autre<br>Refuse de répondre	1<br>2<br>3<br>4<br>5<br>777<br>999	
**104.**	Quelle est votre marque préférée de préservatifs?<br><br>NE PAS LIRE LES REPONSES A HAUTE VOIX - CHOISIR TOUTES LES REPONSES QUI S'APPLIQUENT	Durex<br>Prudence<br>Manix<br>Hot Rubber<br>Protector/USAID<br>Inotek<br>Karma Sutra<br>Le Soft<br>Fresh Feeling<br>Belle Vie<br>Domino<br>Bravo<br>Autre<br>Refuse de répondre	1<br>2<br>3<br>4<br>5<br>6<br>7<br>8<br>9<br>10<br>11<br>12<br>777<br>999	

No.	QUESTIONS ET ELEMENTS DE FILTRAGE DES QUESTIONS	CATEGORIES DE CODAGE		SAUTER LES QUESTIONS
**105.**	Avez-vous utilisé des préservatifs gratuits dans les 6 derniers mois?	Oui<br>Non<br>Je n'ai jamais reçu des préservatifs gratuitement<br>Refuse de répondre	1<br>2<br>3<br><br>999	Si 2, 3, 999 sautez à Q107.
**106.**	Pensez-vous que le fait d'obtenir des préservatifs gratuits augmente la probabilité que vous utilisez un préservatif lors de vos rapports sexuels ?	Oui<br>Non<br>Ne sait pas<br>Refuse de répondre	1<br>2<br>888<br>999	
**107.**	Dans les 6 derniers mois, avez-vous une fois été incapable de vous procurer un préservatif lorsque le besoin s'est manifesté ?	Oui, j'ai été incapable<br>Non, j'ai été toujours capable<br>Refuse de répondre	1<br>2<br>999	Si 2 ou 999 sautez à Q110.
**108.**	Pourquoi ne pouviez-vous pas vous procurer un préservatif chaque fois que vous en aviez besoin?<br><br>NE PAS LIRE LES REPONSES A HAUTE VOIX - CHOISIR TOUTES LES REPONSES QUI S'APPLIQUENT	Il coûte trop cher<br>Le lieu de vente est trop loin<br>Le lieu de vente était fermé<br>Je suis embarrassé par l'achat des préservatifs<br>Je ne sais pas où me procurer des préservatifs<br>Autre<br>Ne sait pas<br>Refuse de répondre	1<br>2<br>3<br>4<br><br>5<br><br>777<br>888<br>999	
**109.**	Au cours des 6 derniers mois, quelles étaient les raisons pour lesquelles vous n'aviez pas obtenu des préservatifs quand vous en aviez besoin?<br><br>NE PAS LIRE LES REPONSES A HAUTE VOIX - CHOISIR TOUTES LES REPONSES QUI S'APPLIQUENT	On peut toujours se procurer des préservatifs<br>Coûte trop cher<br>Pas pratique<br>Clinique ne leur donne pas<br>Embarrassé à obtenir des préservatifs<br>Je ne sais pas où se procurer des préservatifs<br>Autre<br>Refuse de répondre	1<br><br>2<br>3<br>4<br>5<br>6<br><br>777<br>999	
**110.**	Au cours des 6 derniers mois, avez-vous reçu des informations sur l'utilisation du préservatif et des rapports sexuels protégés? Par exemple, grâce à un programme de sensibilisation ou une clinique de santé.	Oui<br>Non<br>Refuse de répondre	1<br>2<br>999	
**111.**	Au cours des 6 derniers mois, avez-vous eu une rupture de préservatif lors de rapports sexuels anaux avec un homme?	Oui<br>Non<br>Refuse de répondre	1<br>2<br>999	Sautez si Q71=0, 888 or 999
**112.**	Avez-vous utilisé un lubrifiant au moment où il a éclaté?	Oui<br>Non<br>Refuse de répondre	1<br>2<br>999	Sautez si Q71=0, 888 or 999

No.	QUESTIONS ET ELEMENTS DE FILTRAGE DES QUESTIONS	CATEGORIES DE CODAGE		SAUTER LES QUESTIONS
**113.**	Avez-vous jamais utiliser plus d'un préservatif à la fois?	Oui<br>Non<br>Refuse de répondre	1<br>2<br>999	
**114.**	Pourriez-vous demander à votre partenaire principal du sexe d'utiliser un préservatif si vous vouliez?	Oui<br>Non<br>Refuse de répondre	1<br>2<br>999	
**115.**	Dans quelles circonstances avez-vous tendance à ne pas utiliser des préservatifs lors de rapports anaux? Avec le sexe anal, nous entendons un pénis qui pénètre dans l'anus d'une personne.<br><br>FAITES UN SONDAGE SUR CHAQUE ELEMENT DE LA REPONSE	y n dk nr<br>Quand j'ai bu ou je suis drogué 1 2 888 999<br>Quand j'ai peur de demander à mon partenaire d'utiliser un préservatif ou s'il refuse 1 2 888 999<br>Lors des rapports sexuels avec un partenaire régulier 1 2 888 999<br>Lors des rapports sexuels avec un partenaire non régulier 1 2 888 999<br>Quand je suis le partenaire insertif (en haut) 1 2 888 999<br>Quand je suis le partenaire réceptif (en bas) 1 2 888 999<br>Lorsque la personne n'éjacule pas en moi 1 2 888 999<br>Autre 1 2 888 999		
**116.**	Vous êtes plus susceptibles d'utiliser un préservatif quand un homme insère son pénis dans votre anus (Butt) ou lorsque vous mettez votre pénis dans le sien ou tout aussi probable?	Lorsque son pénis est en moi<br>Quand mon pénis est en lui / elle<br>Indifféremment susceptible<br>Ne sait pas<br>Refuse de répondre	1<br>2<br>3<br>888<br>999	
**Certaines personnes utilisent du gel lubrifiant pendant les rapports anaux. Le gel lubrifiant rend votre pénis ou le pénis de votre partenaire plus glissante et plus facile à insérer dans l'anus. Le gel lubrifiant empêche également le préservatif de se casser. Maintenant je vous poserai quelques questions sur votre utilisation de gel lubrifiant pendant les 6 derniers mois.**				
**117.**	Dans les 6 derniers mois, avez-vous utilisé un lubrifiant lors de vos rapports anaux avec un homme?	Oui<br>Non<br>Ne sait pas<br>Refuse de répondre	1<br>2<br>888<br>999	Sautez si Q71=0, 888 ou 999.<br>Si 2, sautez a Q124
**118.**	Dans les 6 derniers mois, quels lubrifiants avez-vous utilisé lors de rapports anaux?<br><br>FAITES UN SONDAGE SUR CHAQUE ELEMENT DE LA REPONSE	Oui Non NR<br>Salive 1 2 999<br>Petroleum Jelly 1 2 999<br>(Vaseline, Pommade)<br>Lubrifiant à base d'eau 1 2 999<br>(Vendôme, Durex, etc.)<br>Beurre de Karité 1 2 999<br>Lait corporel, Lotion de main 1 2 999<br>Gel vaginal 1 2 999<br>Huile de Bébé 1 2 9 99<br>Beurre, Blue band, Huile de cuisson1 2 999<br>Autre 1 2 999		

No.	QUESTIONS ET ELEMENTS DE FILTRAGE DES QUESTIONS	CATEGORIES DE CODAGE		SAUTER LES QUESTIONS
**119.**	Dans les 6 derniers mois, où trouviez-vous généralement les lubrifiants à base d'eau ?<br><br>NE PAS LIRE LES REPONSES A HAUTE VOIX - CHOISIR TOUTES LES REPONSES QUI S'APPLIQUENT	Boutique ou supermarché<br>Pharmacie ou une clinique<br>ONG / organisation<br>Amis<br>Partenaire sexuel<br>Autre<br>Refuse de répondre	1<br>2<br>3<br>4<br>5<br>777<br>999	Sautez si le répondant choisit 'Non' pour 'Lubrifiant à base d'eau' en Q118.
**120.**	Êtes-vous en capable d'obtenir des lubrifiants à base d'eau quand vous en avez besoin?	Oui<br>Non<br>Ne sait pas<br>Refuse de répondre	1<br>2<br>888<br>999	Sautez si le répondant choisit 'Non' pour 'Lubrifiant à base d'eau' en Q118.
**121.**	Dans les 6 derniers mois, combien de fois avez-vous utilisé un lubrifiant à base d'eau pour les rapports anaux?<br><br>LIRE LES REPONSES A HAUTE VOIX. COCHEZ UNE SEULE REPONSE	Toujours<br>Quelque fois<br>Jamais<br>Refuse de répondre	1<br>2<br>3<br>999	Sautez si le répondant choisit 'Non' pour 'Lubrifiant à base d'eau' en Q118.<br><br>Sautez si Q71=0, 888 ou 999.
**122.**	Quelle est la raison principale pour laquelle vous n'utilisez pas toujours un lubrifiant à base d'eau pour les rapports anaux?<br><br>NE PAS LIRE LES REPONSES. COCHEZ UNE SEULE REPONSE	Je ne peux pas les obtenir facilement / trop cher<br>Je n'aime pas les lubrifiants<br>Partenaire ne les aime pas<br>Je n'ai jamais entendu parler de ça<br>J'ai honte / gêné pour acheter car il est associé à des homosexuels<br>Autre<br>Ne sait pas<br>Refuse de répondre	1<br>2<br>3<br>4<br>5<br>777<br>888<br>999	Sautez si Q121=1.
**123.**	Au cours des 12 derniers mois, avez-vous reçu des «paquets» de lubrifiant gratuitement? Par exemple, grâce à un programme de sensibilisation ou une clinique de santé.	Oui<br>Non<br>Ne sait pas<br>Refuse de répondre	1<br>2<br>888<br>999	
	H. COMMERCE DU SEXE<br>**Recevoir de l'argent, des biens ou des services en échange des rapports sexuels.**			
	**Maintenant, je vais vous poser quelques questions afin de savoir si vous avez reçu de l'argent, des biens ou des services en échange de rapports sexuels.**			
**124.**	Au cours des 6 derniers mois, qu'est-ce que vous avez reçu en échange du sexe?<br><br>LIRE LES REPONSES A HAUTE VOIX - CHOISIR TOUTES LES REPONSES QUI S'APPLIQUENT	Y N NR<br>Argent 1 2 999<br>Biens 1 2 999<br>Services 1 2 999<br>Autre 1 2 999	1<br>2<br>3<br>888<br>999	Seulement si Q75≠0, 888 ou 999

No.	QUESTIONS ET ELEMENTS DE FILTRAGE DES QUESTIONS	CATEGORIES DE CODAGE		SAUTER LES QUESTIONS
125.	Quel âge aviez-vous quand vous avez eu votre premier rapport sexuel avec une personne en échange d'argent, de biens ou de services?	Année: |__|__| Max: Age du participant (Q7)<br>Ne sait pas<br>Refuse de répondre		Seulement si Q75≠0, 888 ou 999
126.	Quelle est la raison principale pour laquelle vous avez commencé à vendre le sexe pour de l'argent, des biens ou des services?	Besoin d'argent, des biens et des services pour aider la famille<br>Besoin d'argent pour payer une dette contractée<br>Aimer l'avoir / plaisir / l'estime de soi<br>Amis / famille qui le faisaient<br>Autre<br>Ne sait pas<br>Refuse de répondre	1<br>2<br>3<br>4<br>777<br>888<br>999	Seulement si Q75≠0, 888 ou 999
127.	Au cours des 6 derniers mois, avez-vous reçu de l'argent, des biens ou des faveurs en échange de rapports sexuels avec des hommes, des femmes ou les deux sexes?	Hommes<br>Femmes<br>Les deux sexes<br>Refuse de répondre	1<br>2<br>3<br>999	Seulement si Q75≠0, 888 or 999
128.	Depuis combien d'années recevez-vous de l'argent, des biens ou des faveurs en échange de rapports sexuels?<br><br>*(Faites un sondage pour vérifier si la valeur trouvée suppose que la prostitution a commencé avant 13 ans (âge <13 ans)*	Années: |__|__| Moins d'un an<br>Ne sait pas<br>Refuse de répondre	 0<br>888<br>999	Seulement si Q75≠0, 888 ou 999
129.	Dans quelle(s) commune(s) de Bamako exercez-vous le commerce du sexe?<br><br>COCHEZ TOUTES LES REPONSES QUI S'APPLIQUENT	Commune 1<br>Commune 2<br>Commune 3<br>Commune 4<br>Commune 5<br>Commune 6<br>Périphéries<br>Refuse de répondre	1<br>2<br>3<br>4<br>5<br>6<br>7<br>999	Seulement si Q75≠0, 888 ou 999
130.	En général, où trouvez-vous habituellement vos clients de sexe masculin?<br><br>NE PAS LIRE LES REPONSES A HAUTE VOIX<br>COCHEZ TOUTES LES REPONSES QUI S'APPLIQUENT	Ecole / Campus universitaire<br>Concert, Club, Bar, Restaurant<br>Endroit privé<br>Organisation religieuse<br>Centre de gym, de santé ou de beauté<br>Internet<br>Bord de fleuve<br>Dans la rue<br>Autre<br>Refuse de répondre	1<br>2<br>3<br>4<br>5<br>6<br>7<br>8<br>777<br>999	Seulement si Q75≠0, 888 ou 999
131.	Où est-ce que vous avez habituellement des rapports sexuels avec vos clients de sexe masculin?<br><br>NE PAS LIRE LES REPONSES A HAUTE VOIX - CHOISIR TOUTES LES REPONSES QUI S'APPLIQUENT	Ma maison ou celle de quelqu'un d'autre<br>Autre lieu privé<br>Bar/ Hôtel<br>Club<br>Espace ouvert (Parc, Terrain, bord de fleuve, etc.)<br>Lieu de travail<br>Autre<br>Refuse de répondre	1<br>2<br>3<br>4<br>5<br>6<br>777<br>999	Seulement si Q75≠0, 888 ou 999

No.	QUESTIONS ET ELEMENTS DE FILTRAGE DES QUESTIONS	CATEGORIES DE CODAGE		SAUTER LES QUESTIONS
**132.**	Qui décide généralement là où avoir des relations sexuelles?	Vous<br>Client<br>Tous les deux<br>Souteneur<br>Autre<br>Ne sait pas<br>Refuse de répondre	1<br>2<br>3<br>4<br>777<br>888<br>999	Seulement si Q75≠0, 888 ou 999
**133.**	Vendre le sexe est-il votre source de revenu principale?	Oui<br>Non<br>Ne sait pas<br>Refuse de répondre	1<br>2<br>888<br>999	Seulement si Q75≠0, 888 ou 999
**134.**	Combien de clients de sexe masculin aviez-vous eu la semaine passée?	|__|__| Ne sait pas<br>Refuse de répondre	 888<br>999	Seulement si Q75≠0, 888 ou 999.<br>Si 0, allez à Q136.
**135.**	Avec combien de ces *(réponses Q134)* clients avez-vous utilisé des préservatifs ?	|__|__| Max : Q134<br>Ne sait pas<br>Refuse de répondre	 888<br>999	Seulement si Q75≠0, 888 ou 999.
**136.**	Au cours des 6 derniers mois, est-ce qu'un client vous a abusé ou menacé?	Oui<br>Non<br>Ne sait pas<br>Refuse de répondre	1<br>2<br>888<br>999	Seulement si Q75≠0, 888 ou 999
**137.**	Au cours des 6 derniers mois, est-ce qu'un client vous a-t-il forcé à avoir des relations sexuelles?	Oui<br>Non<br>Ne sait pas<br>Refuse de répondre	1<br>2<br>888<br>999	Seulement si Q75≠0, 888 ou 999
I. STIGMATISATION, VIOLENCE ET SANTÉ MENTALE<br>**Maintenant, nous allons vous poser quelques questions sur la stigmatisation, la violence sexuelle et la santé mentale. Rappelez-vous que tout ce que vous direz est confidentiel.**				
**138.**	Pensez-vous que c'est illégal d'être un homme ayant des rapports sexuels avec un homme au Mali ?	Oui c'est illégal<br>Non ce n'est pas illégal<br>Je ne sais pas<br>Refuse de répondre	1<br>2<br>888<br>999	
**139.**	Avez-vous déjà été arrêté parce que vous avez des relations sexuelles avec d'autres hommes?	Oui<br>Non<br>Ne sait pas<br>Refuse de répondre	1<br>2<br>888<br>999	
**140.**	Est-ce que vos amis ou votre famille vous a laissé parce que vous avez des rapports sexuels avec les hommes?	Oui<br>Non<br>Ne sait pas<br>Refuse de répondre	1<br>2<br>888<br>999	
**141.**	Avez-vous été licencié d'un emploi parce que vous avez des relations sexuelles avec d'autres hommes?	Oui<br>Non<br>Ne sait pas<br>Refuse de répondre	1<br>2<br>888<br>999	

No.	QUESTIONS ET ELEMENTS DE FILTRAGE DES QUESTIONS	CATEGORIES DE CODAGE		SAUTER LES QUESTIONS
**142.**	Avez-vous déjà été victime de chantage par quelqu'un parce que vous avez des relations sexuelles avec d'autres hommes?	Oui<br>Non<br>Ne sait pas<br>Refuse de répondre	1<br>2<br>888<br>999	
**143.**	Avez-vous déjà été traité injustement ou privé des soins de santé parce que vous avez des relations sexuelles avec d'autres hommes?	Oui<br>Non<br>Ne sait pas<br>Refuse de répondre	1<br>2<br>888<br>999	
**144.**	Lorsque vous cherchez des soins de santé, est-ce que vous vous sentez dans le besoin de cacher que vous avez des relations sexuelles avec d'autres hommes?	Oui<br>Non<br>Ne sait pas<br>Refuse de répondre	1<br>2<br>888<br>999	
**145.**	Avez-vous déjà subi un harcèlement ou un abus, parce que vous avez des rapports sexuels avec d'autres hommes?	Oui<br>Non<br>Refuse de répondre	1<br>2<br>999	Si 2, allez à Q148.
**146.**	Quel genre de harcèlement ou d'abus était-ce?<br><br>FAITES UN SONDAGE SUR CHAQUE ELEMENT DE LA REPONSE	Y N NR<br>PHYSIQUE (battu) 1 2 999<br>VERBALE (menaces, insultes) 1 2 999<br>MORAL (isolement, exclusion) 1 2 999<br>SEXUEL (forcé à avoir des rapports sexuels) 1 2 999<br>Autre 1 2 999		
**147.**	Qui vous a harcelé ou abusé de vous de cette façon?<br><br>FAITES UN SONDAGE SUR CHAQUE ELEMENT DE LA REPONSE	Oui Non NR<br>Famille 1 2 999<br>Partenaire sexuel 1 2 999<br>Amis, connaissance 1 2 999<br>'AUTORITE' (Leader religieux, employeur, Professeur) 1 2 999<br>Agent de santé 1 2 999<br>Etrangers 1 2 999<br>Détenus de prisons 1 2 999<br>Personnes en uniformes 1 2 999<br>Autre 1 2 999		
**148.**	Avez-vous déjà été forcé à avoir des relations sexuelles contre votre volonté?	Oui<br>Non<br>Refuse de répondre	1<br>2<br>999	Si 2 sautez à Q150.

No.	QUESTIONS ET ELEMENTS DE FILTRAGE DES QUESTIONS	CATEGORIES DE CODAGE		SAUTER LES QUESTIONS
**149.**	Qui vous a forcé à avoir des rapports sexuels contre votre volonté ?<br><br>NE PAS LIRE LES REPONSES A HAUTE VOIX - CHOISIR TOUTES LES REPONSES QUI S'APPLIQUENT	Membres de la famille<br>Partenaire sexuel<br>Amis, connaissance<br>Autorité (Leader religieux, employeur, Professeur)<br>Agent de santé<br>Etrangers<br>Détenus de prisons<br>Personnes en uniformes<br>Autre<br>Refuse de répondre	1<br>2<br>3<br>4<br><br>5<br>6<br>7<br>8<br>777<br>999	
**Ensuite, nous allons vous poser quelques questions sur vos sentiments sur votre attirance sexuelle pour les hommes.**				
**150.**	J'ai essayé de cesser d'être attiré par les hommes en général.	Fortement en désaccord<br>En désaccord<br>Neutre<br>D'accord<br>Tout à fait d'accord<br>Ne sait pas<br>Refuse de répondre	1<br>2<br>3<br>4<br>5<br>888<br>999	
**151.**	Si quelqu'un m'a offert la chance d'être complètement hétérosexuel, j'accepterais la chance.	Fortement en désaccord<br>En désaccord<br>Neutre<br>D'accord<br>Tout à fait d'accord<br>Ne sait pas<br>Refuse de répondre	1<br>2<br>3<br>4<br>5<br>888<br>999	
**152.**	Je voudrais ne pas être gay / bisexuel.	Fortement en désaccord<br>En désaccord<br>Neutre<br>D'accord<br>Tout à fait d'accord<br>Ne sait pas<br>Refuse de répondre	1<br>2<br>3<br>4<br>5<br>888<br>999	
**153.**	Je pense qu'être gay / bisexuel est une lacune personnelle pour moi.	Fortement en désaccord<br>En désaccord<br>Neutre<br>D'accord<br>Tout à fait d'accord<br>Ne sait pas<br>Refuse de répondre	1<br>2<br>3<br>4<br>5<br>888<br>999	
**154.**	Je voudrais obtenir une aide professionnelle afin de changer mon orientation sexuelle gay / bisexuel pour être hétérosexuel.	Fortement en désaccord<br>En désaccord<br>Neutre<br>D'accord<br>Tout à fait d'accord<br>Ne sait pas<br>Refuse de répondre	1<br>2<br>3<br>4<br>5<br>888<br>999	
**COHESION SOCIAL**<br>**Les prochaines questions portent sur votre vie sociale et vos relations avec les autres hommes qui ont des rapports sexuels avec des hommes. S'il vous plaît, écris si vous êtes fortement en désaccord, en désaccord, neutre, d'accord ou fortement d'accord avec les déclarations.**				

No.	QUESTIONS ET ELEMENTS DE FILTRAGE DES QUESTIONS	CATEGORIES DE CODAGE		SAUTER LES QUESTIONS
**155.**	Vous pouvez compter sur d'autres MSM si vous avez besoin d'emprunter de l'argent.	Fortement en désaccord<br>En désaccord<br>Neutre<br>D'accord<br>Tout à fait d'accord<br>Ne sait pas<br>Refuse de répondre	1<br>2<br>3<br>4<br>5<br>888<br>999	
**156.**	Vous pouvez compter sur d'autres MSM pour vous accompagner chez le médecin ou à l'hôpital.	Fortement en désaccord<br>En désaccord<br>Neutre<br>D'accord<br>Tout à fait d'accord<br>Ne sait pas<br>Refuse de répondre	1<br>2<br>3<br>4<br>5<br>888<br>999	
**157.**	Vous pouvez compter sur d'autres MSM si vous avez besoin de parler de vos problèmes.	Fortement en désaccord<br>En désaccord<br>Neutre<br>D'accord<br>Tout à fait d'accord<br>Ne sait pas<br>Refuse de répondre	1<br>2<br>3<br>4<br>5<br>888<br>999	
**158.**	Vous pouvez compter sur d'autres MSM si vous avez besoin de rester quelque part.	Fortement en désaccord<br>En désaccord<br>Neutre<br>D'accord<br>Tout à fait d'accord<br>Ne sait pas<br>Refuse de répondre	1<br>2<br>3<br>4<br>5<br>888<br>999	
**159.**	Au cours des 6 derniers mois, avez-vous négocié avec ou défendu contre un non-MSM afin d'aider un camarade MSM?	Oui<br>Non<br>Ne sait pas<br>Refuse de répondre	1<br>2<br>888<br>999	
J. CONNAISSANCE, OPINIONS ET ATTITUDES FACE AU VIH/SIDA<br>**Maintenant, nous allons vous poser quelques questions sur vos connaissances générales, vos opinions et vos attitudes vis-à-vis du VIH.**				
**160.**	Est-il possible pour une personne qui a l'air d'être en bonne santé d'avoir le VIH?	Oui<br>Non<br>Ne sait pas<br>Refuse de répondre	1<br>2<br>888<br>999	
**161.**	Peut-on réduire le risque d'avoir le VIH en ayant un seul partenaire sexuel séronégatif qui n'a pas d'autres partenaires?	Oui<br>Non<br>Ne sait pas<br>Refuse de répondre	1<br>2<br>888<br>999	
**162.**	Une personne peut-elle réduire le risque de contracter le VIH en utilisant un préservatif chaque fois qu'elle fait des rapports sexuels?	Oui<br>Non<br>Ne sait pas<br>Refuse de répondre	1<br>2<br>888<br>999	
**163.**	Une personne peut-elle attraper le VIH par les piqûres de moustiques?	Oui<br>Non<br>Ne sait pas<br>Refuse de répondre	1<br>2<br>888<br>999	

No.	QUESTIONS ET ELEMENTS DE FILTRAGE DES QUESTIONS	CATEGORIES DE CODAGE		SAUTER LES QUESTIONS
164.	Une personne peut-elle attraper le VIH en partageant un repas avec quelqu'un qui est infecté?	Oui<br>Non<br>Ne sait pas<br>Refuse de répondre	1<br>2<br>888<br>999	
165.	Pensez-vous qu'il est plus probable pour quelqu'un de contracter le VIH lors de rapports sexuels avec des hommes ou des femmes?	Hommes<br>Femmes<br>À propos de la même<br>Ne sait pas<br>Refuse de répondre	1<br>2<br>3<br>888<br>999	
166.	Si un préservatif n'est pas utilisé, quelle voie sexuelle présente le plus grand risque pour contracter le VIH?<br><br>COCHEZ UNE SEULE REPONSE	Le sexe par voie manuelle<br>Le sexe par voie orale<br>Le sexe par voie vaginale<br>Le sexe par voie anale<br>Ne sait pas<br>Refuse de répondre	1<br>2<br>3<br>4<br>888<br>999	
167.	Si un préservatif n'est pas utilisé, quelle voie sexuelle présente le deuxième plus grand risque pour contracter le VIH?<br><br>COCHEZ UNE SEULE REPONSE	Le sexe par voie manuelle<br>Le sexe par voie orale<br>Le sexe par voie vaginale<br>Le sexe par voie anale<br>Ne sait pas<br>Refuse de répondre	1<br>2<br>3<br>4<br>888<br>999	
168.	Comparé au rapport sexuel vaginal, combien important est-il d'utiliser les condoms pour le rapport sexuel anal?<br><br>COCHEZ UNE SEULE REPONSE	Moins important<br>Aussi important<br>Plus important<br>Ne sait pas<br>Refuse de répondre	1<br>2<br>3<br>888<br>999	
169.	Si un préservatif n'est pas utilisé, quel genre de rapport anal vous expose à un plus grand risque pour contracter le VIH?<br><br>COCHEZ UNE SEULE REPONSE	Sexe anal actif (Au-dessus)<br>Sexe anal passif (En dessous)<br>Les deux ont le même risque<br>Les deux n'ont pas de risque<br>Ne sait pas<br>Refuse de répondre	1<br>2<br>3<br>4<br>888<br>999	
170.	Connaissez-vous quelqu'un qui vit avec le VIH ou qui a le sida?	Oui<br>Non<br>Ne sait pas<br>Refuse de répondre	1<br>2<br>888<br>999	Si 2, sautez à Q172.
171.	Connaissez-vous des hommes qui sont VIH positifs et qui ont des rapports sexuels avec d'autres hommes?	Oui<br>Non<br>Ne sait pas<br>Refuse de répondre	1<br>2<br>888<br>999	
172.	Connaissez-vous des hommes qui ont des rapports sexuels avec des hommes qui sont morts du VIH / sida?	Oui<br>Non<br>Ne sait pas<br>Refuse de répondre	1<br>2<br>888<br>999	
173.	Pensez-vous qu'il est possible que vous ayez le VIH ?	Oui<br>Non<br>Je sais déjà que j'ai le VIH<br>Ne sait pas<br>Refuse de répondre	1<br>2<br>3<br>888<br>999	

No.	QUESTIONS ET ELEMENTS DE FILTRAGE DES QUESTIONS	CATEGORIES DE CODAGE		SAUTER LES QUESTIONS
**174.**	A quelle probabilité pensez-vous que vous serez infecté par le VIH dans la prochaine année?	Très peu probable<br>Assez improbable<br>Assez probable<br>Extrêmement probable<br>Ne sait pas<br>Refuse de répondre	1<br>2<br>3<br>4<br>888<br>999	
**175.**	Y a-t-il un traitement efficace contre le VIH / sida?	Oui<br>Non<br>Ne sait pas<br>Refuse de répondre	1<br>2<br>888<br>999	
**176.**	Etes-vous d'accord avec cette affirmation: *"Je ne suis pas aussi prudent par rapport au VIH et des rapports sexuels parce qu'il y a maintenant un meilleur traitement pour le sida"*	D'accord<br>Pas d'accord<br>Ne sait pas<br>Refuse de répondre	1<br>2<br>888<br>999	
K. INFORMATIONS SUR LE VIH ET LES SERVICES<br>**Maintenant, nous allons vous poser quelques questions sur la façon et le type d'information que vous avez reçu sur le VIH et les informations que vous souhaiteriez recevoir.**				
**177.**	Qui ou quoi a le plus d'influence sur votre comportement sexuel à risque?<br><br>NE PAS LIRE LES REPONSES A HAUTE VOIX - CHOISIR UNE SEULE REPONSE	Amis<br>Famille<br>Partenaire sexuel<br>Normes sociales<br>Religion<br>Matériels de sensibilisation du VIH<br>Agent de santé, prestataire de soins<br>Autre<br>Ne sait pas<br>Refuse de répondre	1<br>2<br>3<br>4<br>5<br>6<br>7<br>777<br>888<br>999	
**178.**	Pensez-vous que les messages que vous avez vus sur le VIH se rapportent aux hommes ayant des rapports avec des hommes?	Oui<br>Non<br>Ne sait pas<br>Refuse de répondre	1<br>2<br>888<br>999	
**179.**	Pensez-vous que les messages que vous avez vus sur le VIH se rapportent à vous?	Oui<br>Non<br>Ne sait pas<br>Refuse de répondre	1<br>2<br>888<br>999	Si 1, 888 or 999 allez à Q181.
**180.**	Pourquoi pensez-vous que ces messages ne vous sont pas destinés ?	Ils ne portent pas sur les MSM<br>Ils ne portent pas sur le rapport anal<br>Il y avait une femme dans le message/sur la photo<br>Ils portent sur les femmes enceintes<br>Autre<br>Ne sait pas<br>Refuse de répondre	1<br>2<br>3<br><br>4<br>777<br>888<br>999	

No.	QUESTIONS ET ELEMENTS DE FILTRAGE DES QUESTIONS	CATEGORIES DE CODAGE		SAUTER LES QUESTIONS
**181.**	D'où souhaitez-vous obtenir des informations sur le VIH?<br><br>NE PAS LIRE LES REPONSES A HAUTE VOIX - CHOISIR TOUTES LES REPONSES QUI S'APPLIQUENT	Radio<br>Télévision<br>Journal<br>Internet<br>Téléphone/SMS<br>Brochure<br>Amis<br>Famille<br>Partenaires sexuels<br>Agents de santé<br>Pair éducateur/Agent de sensibilisation<br>Leader religieux<br>Ne sait pas<br>Refuse de répondre	1<br>2<br>3<br>4<br>5<br>6<br>7<br>8<br>9<br>10<br>11<br>12<br>888<br>999	
**182.**	Quels sont les sujets liés au VIH sur lesquels vous voulez avoir plus d'informations ?<br><br>NE PAS LIRE LES REPONSES A HAUTE VOIX - CHOISIR TOUTES LES REPONSES QUI S'APPLIQUENT	Comment est transmis le VIH<br>Comment prévenir le VIH<br>Comment traiter le VIH<br>Comment utiliser le préservatif<br>Sensibiliser son partenaire sur l'utilisation du préservatif<br>Abstinence<br>Monogamie<br>Injections sans risque<br>Autres<br>Ne sait pas<br>Refuse de répondre	1<br>2<br>3<br>4<br>5<br>6<br>7<br>8<br>777<br>888<br>999	
**Nous allons maintenant vous poser des questions sur les services que les pairs éducateurs ou les agents de sensibilisation peuvent vous donner. Un éducateur est quelqu'un comme vous qui a été formé à la prévention du VIH. Un agent de sensibilisation est une personne employée par un organisme, gouvernement ou un organisme privé, qui pourraient fournir ces mêmes services à des gens comme vous.**				
**183.**	Est-ce qu'un pair éducateur ou des agents de sensibilisation ont jamais parlé de vous par rapport au VIH?	Oui<br>Non<br>Ne sait pas<br>Refuse de répondre	1<br>2<br>888<br>999	Si la réponse est 2, sautez à la question 186.
**184.**	Il y a combien de temps qu'un pair éducateur ou un agent de sensibilisation vous a parlé au sujet du VIH?	Dans les 30 derniers jours<br>Dans les 3 derniers mois<br>Dans la dernière année<br>Plus d'un an<br>Ne sait pas<br>Refuse de répondre	1<br>2<br>3<br>4<br>888<br>999	
**185.**	Qu'est-ce que vous avez reçu la dernière fois que vous avez rencontré un pair éducateur ou un agent de sensibilisation?<br>CHOISIR TOUTES LES REPONSES QUI S'APPLIQUENT.	Rien<br>Préservatifs<br>Lubrifiants<br>Dépliant ou une brochure<br>Médicaments<br>Autre<br>Refuse de répondre	1<br>2<br>3<br>4<br>5<br>777<br>999	
L. DEPISTAGE VIH ET PRISE EN CHARGE<br>**Maintenant, je vais vous poser quelques questions sur votre expérience du test de dépistage VIH et de la prise en charge. Rappelez-vous que vos réponses sont confidentielles.**				
**186.**	Avez-vous déjà fait un test de dépistage VIH?	Oui<br>Non<br>Refuse de répondre	1<br>2<br>999	Si 1, allez à la Q188.<br>Si 999, allez à la Q216.

No.	QUESTIONS ET ELEMENTS DE FILTRAGE DES QUESTIONS	CATEGORIES DE CODAGE		SAUTER LES QUESTIONS
**187.**	Quelle est la raison principale pour laquelle vous n'aviez jamais fait un test de VIH?	Je me sens que je ne suis pas à risque pour le VIH<br>Peur de résultat positif<br>Pas d'argent pour faire le test<br>Pas le temps de faire le test<br>La stigmatisation par les travailleurs de la santé<br>Autre<br>Refuse de répondre	1<br><br>2<br>3<br>4<br>5<br><br>777<br>999	Allez à Q216.
**188.**	Quand avez-vous fait votre dernier test du VIH?	Au cours des 6 derniers mois<br>Il y a entre 7 et 12 mois<br>Il y a plus de 12 mois<br>Ne sait pas<br>Refuse de répondre	1<br>2<br>3<br>888<br>999	
**189.**	Pourquoi avez-vous décidé de faire votre tout dernier test de dépistage ?<br><br>NE PAS LIRE LES REPONSES A HAUTE VOIX - CHOISIR TOUTES LES REPONSES QUI S'APPLIQUENT	Parce que je voulais connaitre mon statut<br>J'étais malade/suspicion<br>Nouveau partenaire<br>Je connais quelqu'un nouvellement infecté<br>Sur les conseils de l'agent de santé<br>Pression de l'employeur<br>Dépistage avant le mariage<br>Autre<br>Refuse de répondre	1<br>2<br>3<br>4<br>5<br>6<br>7<br>777<br>999	
**190.**	Lequel des énoncés suivants décrit le mieux le conseil que vous avez reçu?	Respectueux, attentionné, compréhensif<br>Irrespectueux, insensible, stigmatisant, mal à l'aise<br>Ni respectueux ni irrespectueux<br>Ne sait pas<br>Refuse de répondre	1<br>2<br><br>3<br>888<br>999	
**191.**	Avez-vous reçu le résultat de votre test de dépistage ?	Oui<br>Non<br>Refuse de répondre	1<br>2<br>999	Si 2 ou 999 sautez à Q195.
**192.**	A qui avez-vous parlé du résultat de votre test le plus récent?<br><br>NE PAS LIRE LES REPONSES A HAUTE VOIX - CHOISIR TOUTES LES REPONSES QUI S'APPLIQUENT	Personne<br>Partenaire sexuel<br>Membre de la famille<br>Ami<br>Prestataire de soins<br>Autre<br>Refuse de répondre	1<br>2<br>3<br>4<br>5<br>777<br>999	
**193.**	Quel fut le résultat de votre test de dépistage du VIH le plus récent?	Négatif<br>Positif<br>Pas clair / ni positif ni négatif<br>Refuse de répondre	1<br>2<br>3<br>999	Si 2, 3 ou 999 sautez à Q195.
**194.**	Avez-vous déjà été victime de harcèlement ou stigmatisé par d'autres hommes qui ont des rapports sexuels avec des hommes, parce que vous avez le VIH?	Oui<br>Non<br>Refuse de répondre	1<br>2<br>999	Seulement si Q193=2
**Beaucoup de gens positifs consultent un prestataire de soins de santé pour obtenir des soins. Par «soins», nous entendons quelqu'un qui va pour des examens médicaux chez un prestataire de soins de santé ou reçoit des ARV pour son infection par le VIH. Les prochaines questions portent sur la première fois que vous avez vu un prestataire pour votre VIH.**				

No.	QUESTIONS ET ELEMENTS DE FILTRAGE DES QUESTIONS	CATEGORIES DE CODAGE		SAUTER LES QUESTIONS
**195.**	Après avoir testé séropositif, avez-vous consulté un prestataire de soins de santé pour discuter de votre VIH?	Oui<br>Non<br>Ne sait pas<br>Refuse de répondre	1<br>2<br>888<br>999	Si 1, 888, 999 sautez à Q197a
**196.**	Quelle est la raison principale pour laquelle vous n'avez jamais consulté un prestataire de soins de santé pour les soins du VIH?<br><br>COCHEZ UNE SEULE REPONSE	Je me sens en bonne santé<br>La stigmatisation, ne voulant pas que les autres sachent<br>Coût ou des problèmes de transport<br>Mauvaise attitude des travailleurs de la santé<br>Le temps d'attente ou à la clinique heure non convenable<br>Autre<br>Ne sait pas<br>Refuse de répondre	1<br>2<br>3<br>4<br>5<br>777<br>888<br>999	TOUS, sautez à Q216.
**197 a.**	Etes-vous suivi pour la prise en charge VIH au niveau de la Clinique de Halles en 2013?	Oui<br>Non<br>Ne sait pas<br>Refuse de repondre	1<br>2<br>888<br>999	Seulement si Q195=1, 888, 999
**197.**	Avez-vous toujours accédé régulièrement à des soins de santé ou bilans pour votre VIH?	Oui, toujours aux soins<br>Non, j'ai arrêté de recevoir des soins / aller à clinique<br>Ne sait pas<br>Refuse de répondre	1<br>2<br>3<br>888<br>999	Si 1, 888, 999 sautez à Q199
**198.**	Quelle est la raison principale pour laquelle vous n'allez plus à une clinique pour les soins du VIH?<br><br>COCHEZ UNE SEULE REPONSE	Je me sens en bonne santé<br>La stigmatisation, ne voulant pas que les autres sachent<br>coût<br>Mauvaise attitude des travailleurs de la santé<br>Le temps d'attente ou à la clinique heure non convenable<br>Autre<br>Ne sait pas<br>Refuse de répondre	1<br>2<br>3<br>4<br>5<br>777<br>888<br>999	
**Cotrimoxazole ou Cotrim est un médicament recommandé pour les personnes vivant avec le VIH, même si elles n'ont pas commencé le traitement. Il aide à prévenir certaines infections, mais il ne traite pas le VIH. Nous allons vous poser quelques questions sur le cotrimoxazole.**				
**199.**	Avez-vous déjà pris du cotrimoxazole?	Oui<br>Non<br>Ne sait pas<br>Refuse de répondre	1<br>2<br>888<br>999	Si 1 sautez à 201.<br>Si 888, 999 sautez à 202
**200.**	Quelle est la raison principale pour laquelle vous ne prenez pas du cotrimoxazole?	Je me sens en bonne santé<br>La peur de la stigmatisation<br>Non offert par la clinique<br>Clinique trop loin<br>Coût<br>N'est plus efficace<br>Autre<br>Ne sait pas<br>Refuse de répondre	1<br>2<br>3<br>4<br>5<br>6<br>777<br>888<br>999	Tous sautez à Q202
**201.**	Prenez-vous toujours du cotrimoxazole?	Oui<br>Non<br>Ne sait pas<br>Refuse de répondre	1<br>2<br>888<br>999	

No.	QUESTIONS ET ELEMENTS DE FILTRAGE DES QUESTIONS	CATEGORIES DE CODAGE		SAUTER LES QUESTIONS
**Nous allons maintenant vous poser quelques questions au sujet de votre «CD4» ou « nombre de cellules T". Le taux de CD4 indique à quel degré vous êtes malade du VIH ou comment votre système immunitaire est affaibli et si vous avez besoin de prendre des antirétroviraux.**				
**202.**	Avez-vous déjà fait votre test de taux de CD4?	Oui<br>Non<br>Ne sait pas<br>Refuse de répondre	1<br>2<br>888<br>999	Si 2, 888, 999 sautez à Q206
**203.**	Quand est-ce que votre prestataire de soins à testé la dernière fois votre taux de CD4?	Au cours des 6 derniers mois<br>Il y a entre 7 et 12 mois<br>Il y a plus de 12 mois<br>Ne sait pas<br>Refuse de répondre	1<br>2<br>3<br>888<br>999	
**204.**	Avez-vous reçu le résultat du test de CD4 à la même visite ou plus tard?	La même visite<br>Le lendemain<br>Une visite ultérieure<br>Ne sait pas<br>Refuse de répondre	1<br>2<br>3<br>888<br>999	
**205.**	Quel fut le résultat de votre dernier taux de CD4?	Plus de 500<br>Entre 350 et 499<br>Entre 200 et 349<br>En dessous de 200<br>Ne sait pas<br>Refuse de répondre	1<br>2<br>3<br>4<br>888<br>999	
**Nous allons maintenant vous poser quelques questions sur le traitement anti-rétroviral, également appelé ARV, pour traiter le VIH.**				
**206.**	Avez-vous déjà pris des antirétroviraux pour traiter votre VIH?	Oui<br>Non<br>Ne sait pas<br>Refuse de répondre	1<br>2<br>888<br>999	
**207.**	Quelle est la raison principale pour laquelle vous n'avez jamais commencé à prendre des ARV?<br><br>COCHEZ UNE SEULE REPONSE	Mon taux de CD4 est encore élevé<br>Mon taux de CD4 est inconnu<br>Je suis sur une liste d'attente pour commencer<br>Prestataire de soins de santé me dit que c'est trop tôt pour commencer<br>Autre<br>Ne sait pas<br>Refuse de répondre	1<br>2<br>3<br>4<br>777<br>888<br>999	Seulement si Q206=2
**208.**	Vous avez dit autre. Lequel des énoncés suivants est la raison principale pour laquelle vous n'avez pas commencé à prendre des ARV?	Aucune clinique près de chez moi l'offre<br>Le prestataire de soins de santé a refusé de me donner des ARV<br>La peur de la stigmatisation ou que d'autres le sachent<br>Coût trop cher<br>Autre<br>Ne sait pas<br>Refuse de répondre	1<br>2<br>3<br>4<br>777<br>888<br>999	Seulement si Q207=777
**209.**	Prenez-vous actuellement des ARV?	Oui<br>Non<br>Ne sait pas<br>Refuse de répondre	1<br>2<br>888<br>999	Seulement si Q206=1, 888 ou 999
**210.**	Pendant que vous preniez les ARV, est-ce que vous avez utilisé les services suivants?<br><br>COCHEZ TOUT CE QUI S'APPLIQUE	Rappels avec les textes de téléphone mobile<br>Aide de groupe de traitement<br>Aide de nourriture ou d'argent<br>Agent de sensibilisation ou pair éducateur<br>Aucun de ceux-ci<br>Refuse de répondre	1<br>2<br>3<br>4<br>5<br>999	Seulement si Q209=1, 888, ou 999

No.	QUESTIONS ET ELEMENTS DE FILTRAGE DES QUESTIONS	CATEGORIES DE CODAGE		SAUTER LES QUESTIONS
**211.**	Quelle est la raison principale pour laquelle vous avez cessé de prendre des ARV?	Je me sentais en bonne santé<br>Un trop grand nombre d'effets secondaires<br>Dieu me guérira<br>Je ne veux plus prendre les ARV<br>Il était difficile pour moi d'obtenir des ARV<br>Autre<br>Refuse de répondre	1<br>2<br>3<br>4<br>5<br>777<br>999	Seulement si Q209=2
**Les prochaines questions portent sur la tuberculose ou TB. Toutes les personnes vivant avec le VIH doivent être "dépistées" pour la tuberculose. Avec «dépistage», nous entendons le personnel de soins de santé vous pose des questions sur si vous avez une toux, de la fièvre, ou une perte de poids soudaine.**				
**212.**	Avez-vous déjà été dépisté pour la tuberculose?	Oui<br>Non<br>Ne sait pas<br>Refuse de répondre	1<br>2<br>888<br>999	Si 2, 888, 999 sautez à 216
**213.**	Quand est-ce que vous avez été dépisté pour la tuberculose?	Avant que je sois testé séropositif<br>Après avoir été testé séropositif<br>Avant et après avoir été testé séropositif<br>Ne sait pas<br>Refuse de répondre	1<br>2<br>3<br>888<br>999	
**214.**	Quel fut le résultat de votre dernier test de la tuberculose?	Tb-négative<br>Tb-positive<br>Indéterminé<br>Ne sait pas<br>Refuse de répondre	1<br>2<br>3<br>888<br>999	
**215.**	Avez-vous eu un traitement pour la tuberculose?	Oui<br>Non<br>Ne sait pas<br>Refuse de répondre	1<br>2<br>888<br>999	
M. INFECTIONS SEXUELLEMENT TRANSMISSIBLES (IST)<br>**Maintenant, je veux vous poser quelques questions sur votre santé sexuelle. Rappelez-vous que vos réponses sont confidentielles.**				
**216.**	Si vous avez un problème de santé sexuelle demain, où iriez-vous pour vous procurer des soins ?<br><br>NE PAS LIRE LES REPONSES A HAUTE VOIX. CHOISIR UNE SEULE REPONSE.	Pharmacie<br>Hôpital<br>Auto-médication<br>Guérisseur traditionnelle<br>Marabout<br>Soutoura<br>ARCAD/SIDA<br>Clinique Halles<br>Clinique Privée<br>Structure de santé Public<br>Autre<br>Ne sait pas<br>Refuse de répondre	1<br>2<br>3<br>4<br>5<br>6<br>7<br>8<br>9<br>10<br>777<br>888<br>999	

No.	QUESTIONS ET ELEMENTS DE FILTRAGE DES QUESTIONS	CATEGORIES DE CODAGE		SAUTER LES QUESTIONS
**217.**	Au cours des 12 derniers mois, avez-vous consulté un médecin ou une infirmière?	Oui<br>Non<br>Ne sait pas<br>Refuse de répondre	1<br>2<br>888<br>999	Si 2, 888, 999 sautez à Q219
**218.**	Ont-ils vérifié pour voir si vous avez des infections sexuellement transmissibles?	Oui<br>Non<br>Ne sait pas<br>Refuse de répondre	1<br>2<br>888<br>999	
**219.**	Avez-vous eu des **écoulements** anormaux **à partir de votre pénis** au cours des 12 derniers mois?<br><br>MONTRER LA PHOTO	Oui<br>Non<br>Ne sait pas<br>Refuse de répondre	1<br>2<br>888<br>999	
**220.**	Avez-vous eu un **ulcère ou une plaie sur ou à coté de votre pénis** au cours des 12 derniers mois?<br><br>MONTRER LA PHOTO	Oui<br>Non<br>Ne sait pas<br>Refuse de répondre	1<br>2<br>888<br>999	
**221.**	Au cours des 12 derniers mois, avez-vous trouvé un ulcère ou une plaie sur ou à coté du pénis ou du vagin de votre partenaire?	Oui<br>Non<br>Ne sait pas<br>Refuse de répondre	1<br>2<br>888<br>999	
**222.**	Avez-vous eu **un ulcère anal ou une plaie** au cours des 12 derniers mois?<br><br>MONTRER LA PHOTO	Oui<br>Non<br>Ne sait pas<br>Refuse de répondre	1<br>2<br>888<br>999	
**223.**	Avez-vous eu **un écoulement anal** au cours des 12 derniers mois?	Oui<br>Non<br>Ne sait pas<br>Refuse de répondre	1<br>2<br>888<br>999	
**224.**	Avez-vous eu **des verrues anales** au cours des 12 derniers mois?<br><br>MONTRER LA PHOTO	Oui<br>Non<br>Ne sait pas<br>Refuse de répondre	1<br>2<br>888<br>999	Si tous les questions 219-224 = 2, sautez à la question T03.
**225.**	Si vous aviez des rapports sexuels alors que vous aviez ces symptômes, avez-vous informé votre/vos partenaire(s) sexuel?<br><br>*Avec le sexe, nous entendons soit le sexe vaginal, soit le sexe anal. Avec le sexe vaginal, nous entendons un pénis pénètre dans le vagin. Avec le sexe anal, nous entendons un pénis pénètre dans l'anus d'une personne.*	Oui<br>Non<br>Ne sait pas<br>Refuse de répondre	1<br>2<br>888<br>999	

No.	QUESTIONS ET ELEMENTS DE FILTRAGE DES QUESTIONS	CATEGORIES DE CODAGE		SAUTER LES QUESTIONS
**226.**	Pendant que vous aviez ces symptômes, êtes-vous abstenu d'avoir des rapports sexuels?	Oui<br>Non<br>Ne sait pas<br>Refuse de répondre	1<br>2<br>888<br>999	
**227.**	Pendant que vous aviez ces symptômes, avez-vous de toujours utilisé un préservatif lors des rapports sexuels?	Oui<br>Non<br>Ne sait pas<br>Refuse de répondre	1<br>2<br>888<br>999	
**228.**	Pourquoi n'avez-vous pas informé votre/vos partenaire(s) que vous pourriez souffrir d'une IST ?<br><br>NE PAS LIRE LES REPONSES<br>CHOISIR UNE SEULE REPONSE	J'avais peur que mon partenaire soit fâché<br>J'avais peur que mon partenaire devienne violent<br>J'avais peur que mon partenaire me quitte<br>J'avais peur que mon partenaire le dise aux autres<br>Je ne savais pas comment localiser mon partenaire<br>Je ne pensais pas que c'était nécessaire d'en discuter<br>J'ai reçu un traitement<br>Autre<br>Refuse de répondre	1<br>2<br>3<br>4<br>5<br>6<br>7<br>777<br>999	Sautez si Q225=1.
**229.**	Est-ce que la personne avec qui vous avez eu des rapports sexuels a reçu un traitement pour ce problème?	Oui<br>Non<br>Ne sait pas<br>Refuse de répondre	1<br>2<br>888<br>999	
**230.**	Avez-vous vu un prestataire de soins de santé à cause de ces problèmes?	Oui<br>Non<br>Ne sait pas<br>Refuse de répondre	1<br>2<br>888<br>999	
**231.**	Etes-vous allé à une pharmacie pour obtenir un traitement?	Oui<br>Non<br>Ne sait pas<br>Refuse de répondre	1<br>2<br>888<br>999	
**232.**	Combien de temps a-t-il fallu pour aller voir le prestataire de soins de santé depuis l'apparition de ces problèmes?<br><br>NE PAS LIRE LES REPONSES<br>COCHEZ UNE SEULE REPONSE	Moins d'une semaine<br>Plus d'une semaine, moins d'un mois<br>Plus d'un mois<br>Ne sait pas<br>Refuse de répondre	1<br>2<br>3<br>888<br>999	Seulement si Q230=1
**233.**	Pourquoi n'avez-vous pas cherché à vous procurer un traitement pour ce problème?<br><br>NE PAS LIRE LES REPONSES<br>COCHEZ UNE SEULE REPONSE	Je pensais que le mal partirait de lui-même<br>Je ne sais pas où l'obtenir<br>Préoccupé par la confidentialité<br>Attitude négative du personnel de santé<br>Coût<br>Distance<br>Autre<br>Refuse de répondre	1<br>2<br>3<br>4<br>5<br>6<br>777<br>999	Sauter si Q230=1 et Q231=1.

No.	QUESTIONS ET ELEMENTS DE FILTRAGE DES QUESTIONS	CATEGORIES DE CODAGE		SAUTER LES QUESTIONS
**234.**	Est-ce que le professionnel de la santé vous a dit que vous avez eu une infection sexuellement transmissible?	Oui<br>Non<br>Ne sait pas<br>Refuse de répondre	1<br>2<br>888<br>999	Seulement si Q230=1
**235.**	La dernière fois que vous avez eu une infection sexuellement transmissible, vous êtes-vous senti à l'aise avec les soins administrés par l'agent de santé ?	Oui<br>Non<br>Ne sait pas<br>Refuse de répondre	1<br>2<br>888<br>999	Seulement si Q230=1
**236.**	La dernière fois que vous avez été traité pour une infection sexuellement transmissible, vous êtes-vous senti stigmatisé par l'agent de santé?	Oui<br>Non<br>Ne sait pas<br>Refuse de répondre	1<br>2<br>888<br>999	Seulement si Q230=1

Paramètres du Questionnaire			
**T03**	Identifiant de l'étude<br><br>*S'il vous plaît répondez à cette question avant de laisser sortir le participant à l'étude.*<br><br>*A chaque participant à l'étude, il est attribué un numéro d'identification séquentiel à l'étude de quatre chiffres: XZZZ, où «X» désigne le site de l'étude (A ou B) et «ZZZ» est le numéro d'ordre commençant par le participant 001.*	☐☐☐☐	
**T04**	L'interview a été réalisée sans incident?	Oui<br>Non	1<br>2
**T05**	Raison de l'entretien inachevé<br><br>(Si T04=2)	Peur d'être vu ou attrapé<br>Questions gênantes<br>Pas de temps<br>Pas motivé<br>Inéligible<br>Autre<br>Refuse de répondre	1<br>2<br>3<br>4<br>5<br>777<br>999
